# Amplification of MED30 at chromosome 8q24 reprograms MYC binding to low-affinity oncogenic enhancers in cancer cells

**DOI:** 10.1016/j.celrep.2026.117498

**Published:** 2026-06-11

**Authors:** Chunyu Jin, Linjie Zhao, Wubin Ma, Guofeng Zhao, Yujia Liu, Hanwen Zhang, Shenghong Ma, Likun Yao, Yuan Liu, Qiulian Wu, Huairui Yuan, Kailin Yang, Wei Yuan, Kenneth Ohgi, Jeremy N. Rich, Michael G. Rosenfeld

**Affiliations:** 1 Department and School of Medicine, San Diego, La Jolla, CA, USA; 2 UPMC Hillman Cancer Center, Pittsburgh, PA, USA; 3 Department of Pharmacology and Moores Cancer Center, San Diego, La Jolla, CA, USA; 4 Department of Bioengineering, University of California, San Diego, La Jolla, CA, USA; 5 Department of Neurology, University of Pittsburgh Medical Center, Pittsburgh, PA, USA; 6 Department of Radiation Oncology, Taussig Cancer Center, Cleveland Clinic, Cleveland, OH, USA; 7 These authors contributed equally; 8 Lead contact

## Abstract

The activity of many oncogenic transcription factors (TFs) is constrained by enhancer binding-site affinity, leaving many low-affinity sites unoccupied under physiological conditions. Whether coactivator amplification in cancer redirects TFs to these sites is unclear. Here, we show that amplification of MED30, a Mediator subunit at 8q24, promotes aberrant MYC binding to low-affinity regulatory regions and is associated with poor outcomes. Besides frequent MYC MED30 co-amplification, MED30 overexpression alone is sufficient to enable MYC occupancy and activation of previously weak or unbound enhancers and promoters, driving tumor-promoting gene expression. Functional studies in pancreatic ductal adenocarcinoma and glioblastoma demonstrate that MED30 is oncogenic and serves as a prognostic marker independent of MYC amplification. These findings reveal a cofactor-driven mechanism by which MED30 licenses MYC binding to low-affinity sites, reprogramming enhancers during cancer progression with therapeutic implications.

## In brief

Jin et al. show that amplification of the mediator subunit MED30 redirects MYC to low-affinity regulatory elements, enabling *de novo* enhancer activation and oncogenic transcription. MED30 overexpression alone is sufficient to reprogram MYC binding and predict poor prognosis, revealing a cofactor-driven mechanism of enhancer rewiring in cancer.

## Graphical abstract

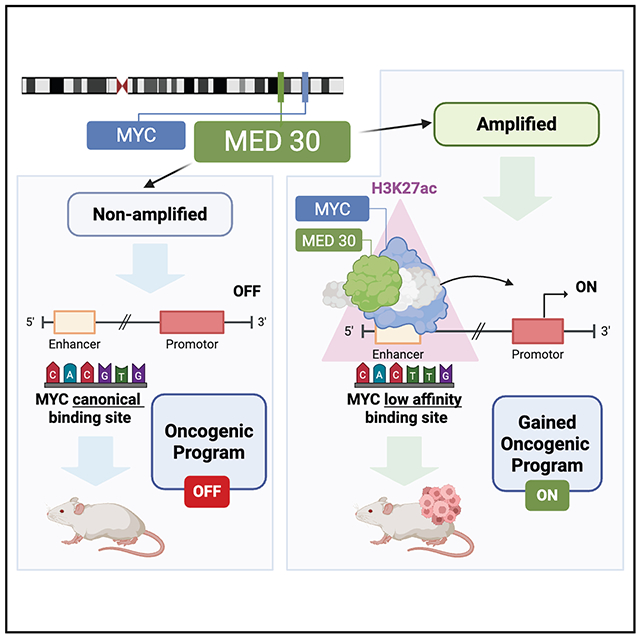

## INTRODUCTION

Enhancer specificity is increasingly recognized to depend on submaximal recognition motifs,^1–4^ emphasizing the importance of understanding the regulatory strategies underlying binding to low-affinity transcription factor (TF) binding promoter sites. Indeed, in developmentally important enhancers, increased strength of TF binding causes serious developmental abnormalities.^5^ Therefore, we posit that gene amplification or overexpression (OE) of TFs and cofactors that would license binding of oncogenic TFs to low affinity enhancer/promoter sites might activate oncogenic programs that would lead to worse clinical outcomes. This could be investigated by taking advantage of data in which amplification of any specific cofactor might be linked to activation of additional oncogenic programs.

Genome-wide sequencing have accumulated large amount of data on genetic variations in human disease, including cancers, which are driven by the simultaneous dysregulation of multiple genes.^6–8^ The *MYC* gene, which encodes the TF c-Myc, is the most frequently amplified oncogene driver in human cancers, and its elevated expression correlates with tumor aggression and poor clinical outcome. The oncogenic effects of MYC depend on the transcription activity in which it binds to E-box sites as a heterodimer with MAX,^9^ and to serve as a global “transcription amplifier” for existing transcribed target genes, underlying the mechanism for rapid proliferation in cancer cells.^10,11^ However, despite considerable study on the actions of *MYC* OE with respect to tumor aggressiveness, it is not yet clear which transcriptional machinery/co-factors are involved in its recruitment and how elevated MYC activity is mechanistically coupled to malignant transcriptional reprogramming. One of the genes in the amplified region encodes a subunit of the mediator coactivator complex, MED30. Additionally, MYC has proven difficult to directly target pharmacologically. Thus, alternative strategies to disrupt its transcriptional activity are being pursued.^12,13^ Identifying key cofactors that facilitate elevated MYC transcriptional activity could provide crucial mechanistic insights and uncover novel therapeutic opportunities.

Mediator (MED) is a 30-subunit complex that plays a central role in transcription regulation, in part by integrating regulatory signals from transcriptional factors to RNA polymerase II (Pol II).^14–20^ The MED complex regulates gene expression at multiple stages of transcription, from promoting assembly of the preinitiation complex (PIC) to facilitating efficient entry into elongation or promoter escape.^14,16,21–24^ The complex contains three main integral modules; referred to as head, middle, and tail, and a dissociable kinase module (CDK8, CycC, MED12, and MED13).^25^ The kinase module has been associated primarily with repressive functions but has also been implicated in activation of transcription (reviewed in Yin and Wang^24^). The critical role of mediator complex in transcriptional regulation and the observation that MED30 is amplified or overexpressed not only in ~70% of cancers exhibiting *MYC* amplification, but also in up to 5% even in the absence of MYC amplification permits exploration if MED30 amplification does license an oncogenic enhancer program. We have uncovered the importance of MED30 gain-of-function on activating such and oncogenic cohort of enhancers, accelerating tumor growth in pancreatic cancer and glioblastoma (GBM) cancers.

## RESULTS

### MED30 and MYC co-amplification across various cancer types

**Figure 1.**
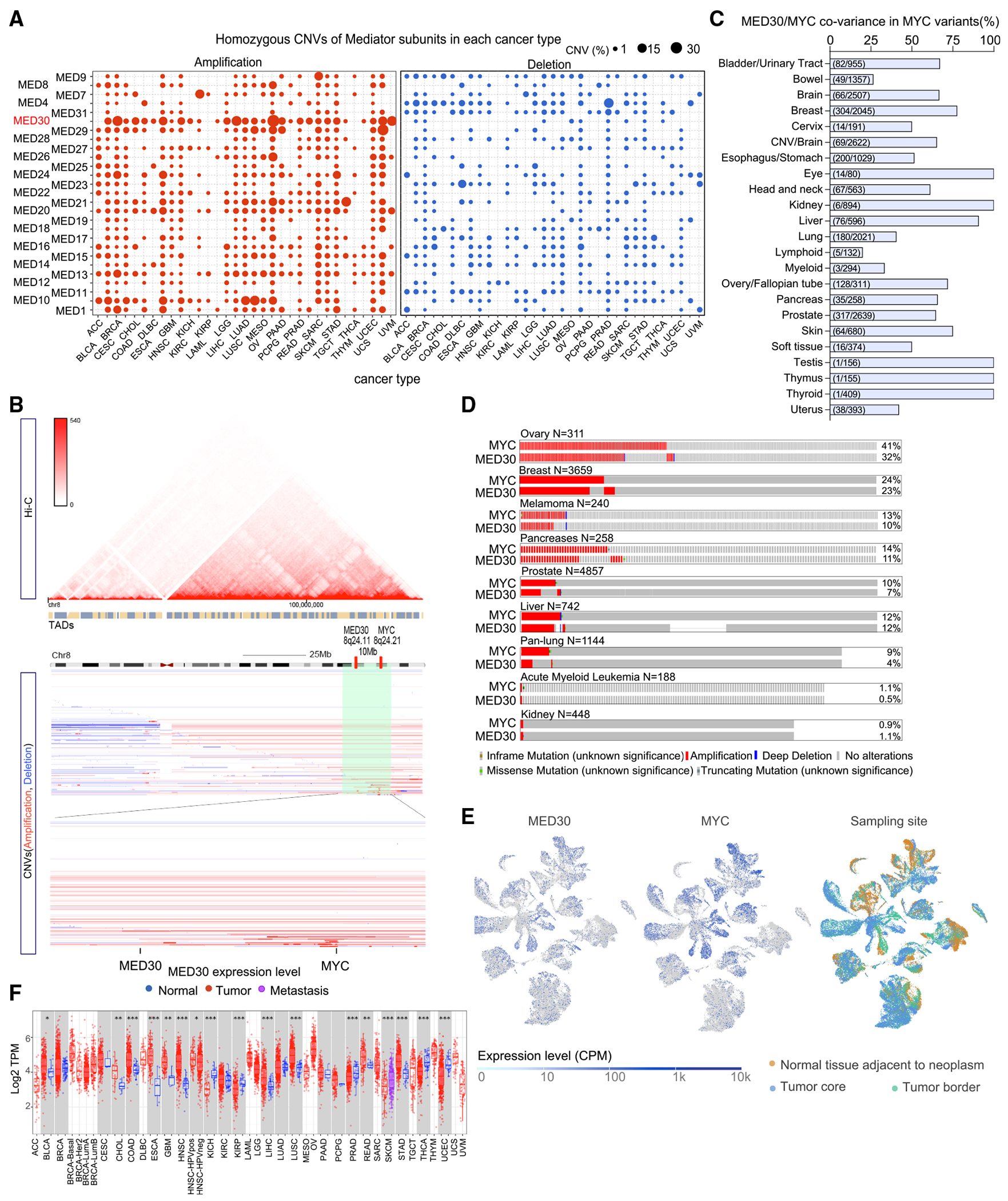
MED30 and MYC gene co-amplification in cancers (A) Gene amplification and deletion frequency of each subunit of mediator complex (data from TIMER). (B) Copy number variances (CNVs) in chromosome 8 in 175 patients/samples in pancreatic adenocarcinoma. Each row represents a single sample (data from TCGA, PanCancer Atlas). Upper: Hi-C interaction map showing MED30 and MYC genomic loci in pancreatic PANC1 cells at 40 kb resolution (data from ENCODE3). (C) Frequency of gene alterations co-occurrence for MED30 and MYC in MYC-altered case. The numbers in column indicate MYC-altered cases out of total number of cases, and the *x* axis percentage indicates the ratio of MYC and MED30 both altered cases out of MYC-altered cases. (D) Gene alteration co-occurrence of MED30 and MYC in indicated patient samples, each vertical line indicates a sample (data from cBioPortal). (E) Umap of single-cell RNA-seq analysis for expression of MYC and MED30. (F) Expression level of MED30 in all cancer types based on TCGA tumor and normal data (Timer2.0). The statistical significance computed by the Wilcoxon test is annotated by the number of stars (*p < 0.05; **p < 0.01; ***p < 0.001).

Genetic alterations such as mutation, amplification, deletion, and translocation are key drivers of tumorigenesis. To identify factors contributing to the aberrant transcriptional states associated with cancer, we examined the role of altered expression of mediator (MED) complex components across diverse cancer types. Using *in silico* analysis of the cBioPortal cancer genomics database (https://www.cbioportal.org), which includes data from approximately 10,000 patients spanning dozens of cancer types, we found that the majority of MED gene variants were amplification events. The high amplification frequency of specific MED subunits suggests a gain-of-function role in cancer development. Although the prevalence of MED gene alterations varied by cancer type, the overall pattern of alteration was broadly conserved, indicating shared oncogenic functions of the mediator complex. To further investigate MED gene copy number alterations across pan-cancer datasets, we analyzed amplification and deletion frequencies using the Gene Set Cancer Analysis (GSCA) database. These results were consistent with those from cBioPortal, revealing that MED30, which encodes a small 178-amino acid mediator subunit, exhibited the highest amplification frequency among all MED genes. MED30 was amplified in over 10% of cases in half of all cancer types (Figure 1A).

The MED30 gene is located on chromosome 8q24, one of the most frequently amplified regions across multiple cancer types.^26^ This locus harbors several genes and cancer-associated genetic variants, the most notable being MYC.^27,28^ MED30 lies approximately 10 megabases upstream of the MYC locus, and this close genomic proximity likely contributes to their frequent co-amplification (Figure 1B). To assess the prevalence of this co-amplification, we analyzed data across all cancer types and found that, on average, 70% of MYC-amplified cases also exhibit MED30 amplification (Figure 1C). Among various alterations affecting these two genes, co-amplification was the most common, while deletions were rare (Figures 1D, S1A, and S1B). In line with this genomic correlation, MED30 and MYC mRNA expression levels were also strongly correlated at the single-cell level, as demonstrated by an analysis of 52,609 single-cell RNA-seq profiles from colorectal tumors and adjacent non-malignant colon tissues^29^ (Figure 1E). Moreover, MED30 expression was consistently higher in tumor cells compared to normal cells across many cancer types (Figure 1F). Together, these genetic and transcriptomic data support a strong co-amplification and co-expression relationship between MED30 and MYC in cancer.

### MED30 OE shifts the MYC transcriptional program

Pancreatic ductal adenocarcinoma (PDAC) is a genetic disease driven by gene alterations, such as KRAS mutation, CDKN2A, TP53, and SMAD4 inactivation, as well as GATA6 and MYC amplification.^30^ MYC amplification promotes pancreatic cancer progression by acting as a TF that regulates genes involved in metabolism, hypoxia, and proliferation, and is correlated with poor clinical outcome.^31^ Moreover, MYC is a major contributor to PDAC heterogeneity, with metastatic progression linked to activation of MYC signaling pathways and a notable enrichment for MYC amplification in metastatic patients.^32^ Given the mortality of PDAC and the pivotal role of MYC amplification, we investigated the cooperative function of MYC and MED30 in this cancer type.

**Figure 2.**
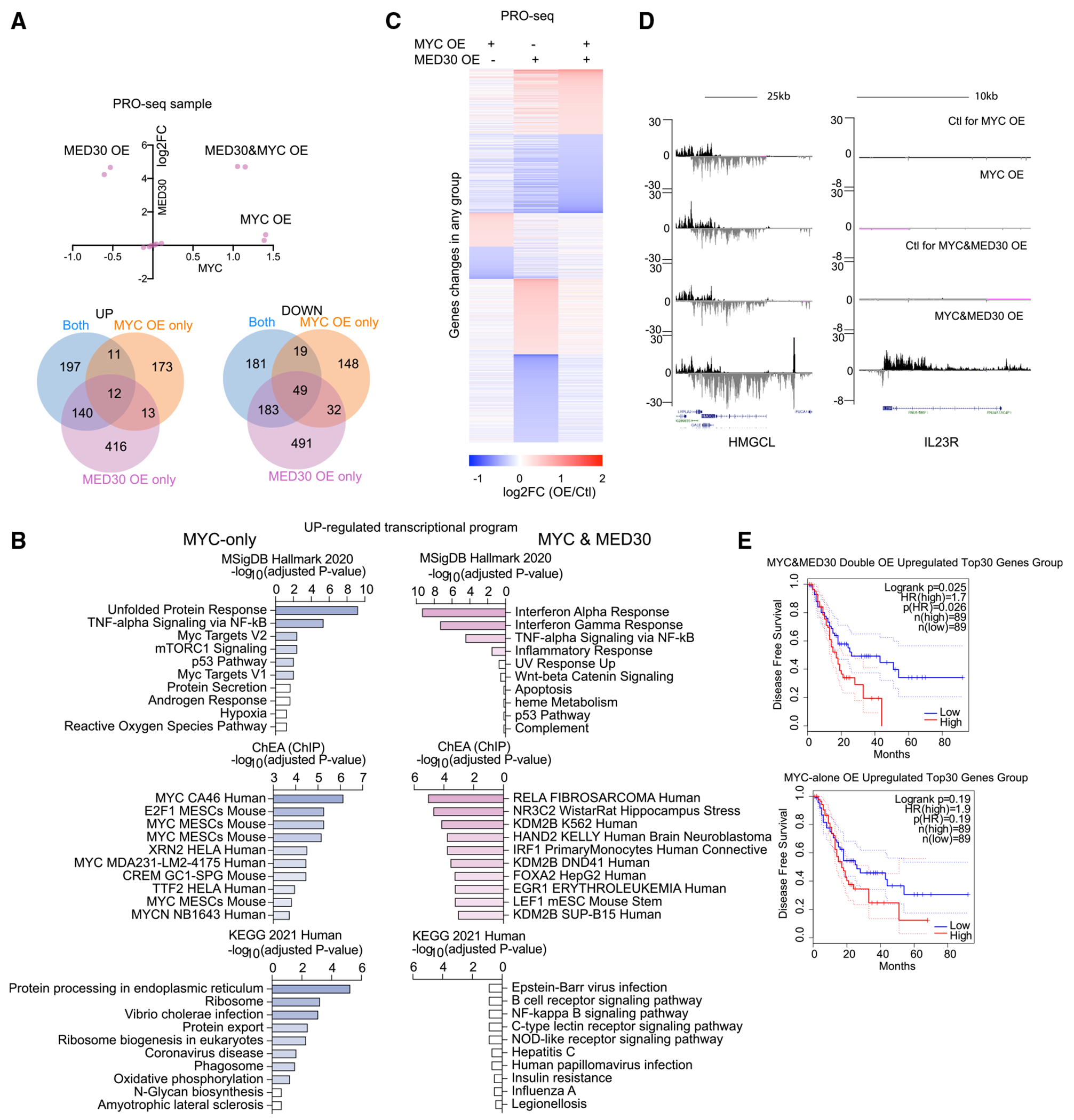
Transcriptional alteration of MED30, MYC or double OE in Mia PaCa-2 pancreatic cancer cells (A) Upper: log2 fold change of MYC or MED30 gene in PRO-seq samples. Lower: overlap of regulated genes in the three indicated groups in PRO-seq. (B) Enrichment analysis for MYC-only OE or MYC&MED30 co-OE upregulated genes. (C) Heatmap transcription level log2 fold change of indicated OE vs. non-OE. Red and blue indicate increased and decreased transcription, respectively. Genes are clustered based on fold-change. (D) UCSC genome browser tracks of PRO-seq data at example gene loci: HMGCL and IL23R are regulated by MYC&MED30 double OE but not MYC-alone OE. (E) Pancreatic cancer patients disease-free survival analysis for high and low expression of top30 upregulated genes by MYC-alone OE and MYC&MED30 double OE.

To examine the transcription regulation driven by *MYC* or *MED30* amplification alone versus combined MYC&MED30 co-amplification, we performed precision run-on sequencing (PRO-seq) in Mia PaCa-2 cells engineered for doxycycline-inducible OE of MYC (HA-tagged), MED30, or both. Each condition has a control that was not treated with doxycycline. The three groups all have good reproducibility within replicates and separation between treatment (Figure S2A). Induction with 0.5 μg/mL doxycycline effectively triggered expression of the respective transgenes, mimicking physiological states of single and co-amplification (Figures 2A and S2B). Despite PRO-seq analysis revealed a modest reduction in nascent MYC transcription upon MED30 OE; this did not result in a detectable change in steady-state MYC mRNA (Figure S2C). Two-day induction resulted in upregulation of 209 genes for MYC OE, 587 genes for MED30 OE and 360 genes for MYC&MED30 OE (log2FC > 0.35, *p* < 0.05) and downregulation of 248 genes for MYC OE, 755 genes for MED30 OE, and 432 genes for MYC&MED30 OE (log2FC < −0.35, *p* < 0.05) (Figure 2A). Small changes in nascent transcription can produce substantial downstream effects through transcriptional amplification. The genes upregulated by *MYC* OE were enriched for canonical *MYC* targets (Hallmark MYC targets V1 and V2^33^), validating that induced exogenous MYC works equivalently to endogenous MYC (Figure 2B). Interestingly, comparative analysis revealed that the transcriptional program induced by *MYC*/*MED30* co-OE was distinct from that of either factor alone, yet more closely resembled the *MED30*-driven program (Figure 2C). This suggests many of the MYC+MED30 regulated genes are also similarly regulated under *MED30* OE condition and are less altered in the MYC-alone OE.

Because mediator and MYC primarily function as transcription activators, the upregulated transcription program is likely to be the direct target in *MYC* and/or *MED30* OE. Functional pathway enrichment analysis revealed that the upregulated genes in MYC-only OE were predominantly involved in the protein processing and ribosome biogenesis, which is well-established functions of MYC.^34^ In contrast, this pattern was not observed in MYC&MED30 co-OE condition. Instead, the top enriched pathway in *MYC&MED30* co-OE upregulated genes was related to inflammation, such as TNF-alpha/RELA (Figure 2B). Notably, some well-characterized oncogenes were specifically upregulated under *MYC&MED30* co-OE condition, but not in MYC-only OE. These include *HMGCL*, critical for tumorigenesis and progression in spontaneous pancreatic cancer model in mice^35^; and the *IL-23 receptor* (*IL-23*R), initially identified in tumor-infiltrating immune cells and also expressed in tumor cells.^36,37^ The expression of *IL-23R* in tumor cells is associated with a poor prognosis, potentially due to IL-23 binding to its receptor and promoting tumor migration and invasion.^38^ These genes are all found significantly upregulated with MYC&MED30 co-OE, but not with *MYC* OE alone (Figure 2D). Furthermore, the top 30 significant upregulated genes from *MYC&MED30* co-OE were associated with poor disease-free survival signature in pancreatic cancer patients, whereas MYC-only upregulated genes do not exhibit a statistically significant prognostic effect, at lease with the current data size (GEPIA, gepia.cancer-pku.cn) (Figure 2E). To validate that transcriptional activity observed in PRO-seq correlates with mRNA abundance, we also performed RNA-seq in only *MED30* OE after 2 days of induction (all genes expression level and DESeq2 results are listed in Tables S1 and S2). The RNA-seq data were consistent with the PRO-seq results, further supporting the shift in transcriptional output (Figure S2D). Together, these findings indicate that *MYC&MED30* co-amplification induces a distinct transcriptional program compared to either factor alone, with the *MED30* OE program enhancing oncogenic pathways that may drive more aggressive pancreatic cancer progression.

### Recruitment of MYC by MED30

**Figure 3.**
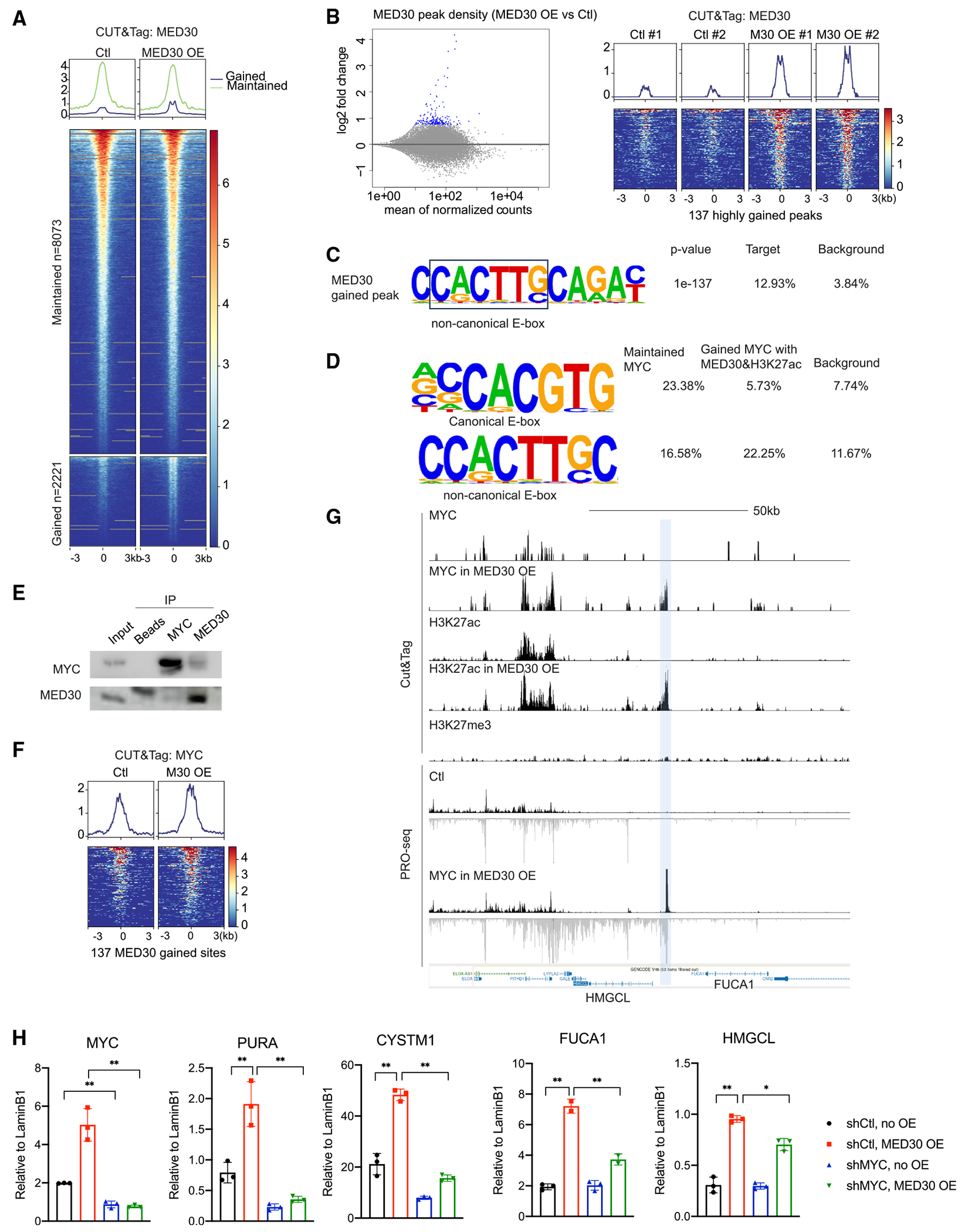
MED30 OE causes additional MYC binding on gene regulatory region and activates adjacent gene expression (A) Read counts density heatmap of MED30 maintained and gained binding peaks in control of MED30 OE condition revealed by CUT&Tag experiments were performed in doxycycline-induced MED30 OE Mia PaCa-2 cells. Dox 0.5 μg/mL 2 days. (B) Left: MA plot for MED30 binding peaks in Dox-induced MED30 OE vs. control. Blue dots labeled the MED30 gained peaks (*n* = 137) and lost peaks (*n* = 127) (|log2FC| > 0.8, *p* < 0.05). Right: tag density heatmap of these 137 gained peaks in control or MED30 OE condition. (C) Homer *de novo* motif result of MED30 gained peaks in MED30 CUT&Tag upon MED30 OE. (D) Comparison of MYC canonical and non-canonical motif enrichment in indicated groups (experiment: MYC CUT&Tag upon MED30 OE). (E) CoIP experiment of MYC and MED30 in Mia PaCa-2 cells. (F) MYC binding density in control of MED30 OE condition in 137 MED30 gained sites, by MYC CUT&Tag experiments in Mia PaCa-2 cells (median signal: 16.38 in control vs. 22.07 in MED30 OE; paired *t* test *p* = 4.09 × 10^−7^). (G) Genome browser examples of MYC new generated bindings site (light blue highlighted) and nearby gene transcription in MED30 OE and control. (H) RT-qPCR to detect MED30-induced genes example with or without MED30 OE or shMYC knockdown in Mia PaCa-2 cells. Data are presented as mean ± SD.**p < 0.01; NS, not significant, two-tailed Student’s *t* test.

To examine whether MED30 OE results in additional bindings sites, or the increase of existing sites, we performed CUT&Tag for MED30 in Tet-on MED30-overexpressing Mia PaCa-2 cells, with or without doxycycline induction for 3 days. The MED30 binding profile clearly distinguished the MED30-overexpressing condition from the control (Figure S3A). The MED30 CUT&Tag results under normal and MED30-overexpressed conditions indicate that OE does not lead to a global increase in peak signals. Instead, changes occur at specific sites, with some peaks showing increases (gains) and others decreases (losses) (Figures 3A and S3B). The number of lost peaks exceeds the number of gained peaks. This pattern is consistent with a reallocation of mediator from a subset of pre-existing binding sites to newly engaged regulatory regions, rather than uniform recruitment to all sites. Among the stable peaks, approximately 60% are located at promoter regions, while about 33% are found in distal intronic or intergenic regions. In contrast, the unstable peaks—those gained or lost—are more frequently located in distal intergenic and intronic regions (~60%) rather than promoters (Figure S3B).

Applying stringent criteria (log_2_FC > 0.8, *p* < 0.05), we identified 137 significantly gained MED30 binding sites and 127 significantly reduced sites (log_2_FC < −0.8, *p* < 0.05) (Figure 3B). Using ENCODE ATAC-seq data in PANC-1 (ENCFF182SSP.bed.gz) as reference, we found 106 of these 137 gained MED30 peaks are located in open chromatin, comparable to 79% of maintained peaks, indicating the gained binding sites are largely located within pre-existing accessible chromatin. The gained peaks are enriched in bHLH binding motif (Figure 3C). As MYC is a bHLH TF, its canonical high-affinity binding site is the E-box sequence CACGTG. Variants of this sequence, typically CANNTG, are widely considered non-canonical or lower-affinity E-box motifs. We performed MYC CUT&Tag with or without doxycycline-induced MED30 OE and identified 2,917 maintained peaks and 908 gained peaks overlap with H3K27ac and MED30. Twenty-three precent of MYC maintained peaks (682/2,917) contain the canonical CACGTG motif, which is the top enriched motif in this group. In contrast, only 5.7% of MED30-associated gained MYC peaks (52/908) contain the canonical motif. Instead, these gained peaks are enriched for the non-canonical E-box variant CACTTG, which is present in 22% of gained peaks (202/908) (Figure 3D). These results indicate that MED30-dependent MYC recruitment preferentially occurs at enhancer regions containing non-canonical, lower-affinity MYC binding motifs rather than the canonical E-box, supporting the concept that MED30 facilitates MYC binding at low-affinity regulatory elements.

The gained MYC binding enhancers correlate with 624 genes (peaks locate in gene region or within 20 kb of transcription start site (TSS)). These genes’ PRO-seq expression level in MED30 OE is upregulated compared to random genes, which are unaltered (Figure S3C) The hallmark pathway enrichment these induced genes revealed “interferon alpha response (*p* = 0.00016),” hypoxia (*p* = 0.00016), and adipogenesis (*p* = 0.00463) are top enriched terms, suggesting this transcriptional program is associated with cancer characteristics (Figure S3D). MYC was detected in co-immunoprecipitation with MED30, suggesting an association between the two proteins. This association may be direct or indirect through other mediator components (Figure 3E). The MED30 significantly gained sites also correlated with corresponding increases in MYC binding (Figure 3F).

We next examined the regulatory consequences of MED30 OE at specific genomic loci. For example, MED30 OE induced MYC binding at a putative intergenic enhancer between the *PURA* and *CYSTM1* promoters (Figure S3E), and expression of both genes was significantly upregulated (Figure S3F). In contrast, *IGIP*, a gene located between *PURA* and *CYSTM1*, remained inactive and unbound by mediator, and its expression was unaffected by *MED30* OE. However, *NRG2*, located nearby in a region marked by the repressive H3K27me3 modification, but also bound by MYC and mediator, exhibited mild activation upon *MED30* induction. A similar regulatory pattern was observed at the *HMGCL-FUCA1* locus, where MED30 OE triggered *de novo* MYC binding at a putative intergenic enhancer and upregulation of both genes (Figure 3G). Notably, MYC knockdown attenuated MED30-induced *FUCA1* expression (Figure 3H). ENCODE H3K4me1 HiChIP datasets generated from an independent cell line human embryonic stem cell-derived pancreatic hormone progenitor cells (H9/WA09) revealed positive chromatin interaction signals linking the putative enhancers with the HMGCL-FUCA and PURA-CYSTM1 gene loci (Figure S3G). We also performed H3K4me1 ChIP-qPCR at selected putative enhancers, H3K4me1 is significantly enriched at these sites upon MED30 OE (Figure S3H). These results support the putative enhancers are indeed enhancer for these genes. To test the specificity of MED30, we express another mediator subunit MED4, and did not find the upregulation of these genes (Figure S3I). This indicates that the observed effect is not a general property of mediator subunit OE but is specific to MED30. Taken together, these results demonstrate that MED30 OE facilitates MYC recruitment to novel regulatory elements, promoting transcriptional activation of target genes. The functional relevance of this recruitment is underscored by the requirement of MYC for the full activation of these genes, highlighting a mechanistic basis for the altered transcriptional program seen in MYC+MED30 co-OE.

### MED30 recruits other mediator components to the gained sites

**Figure 4.**
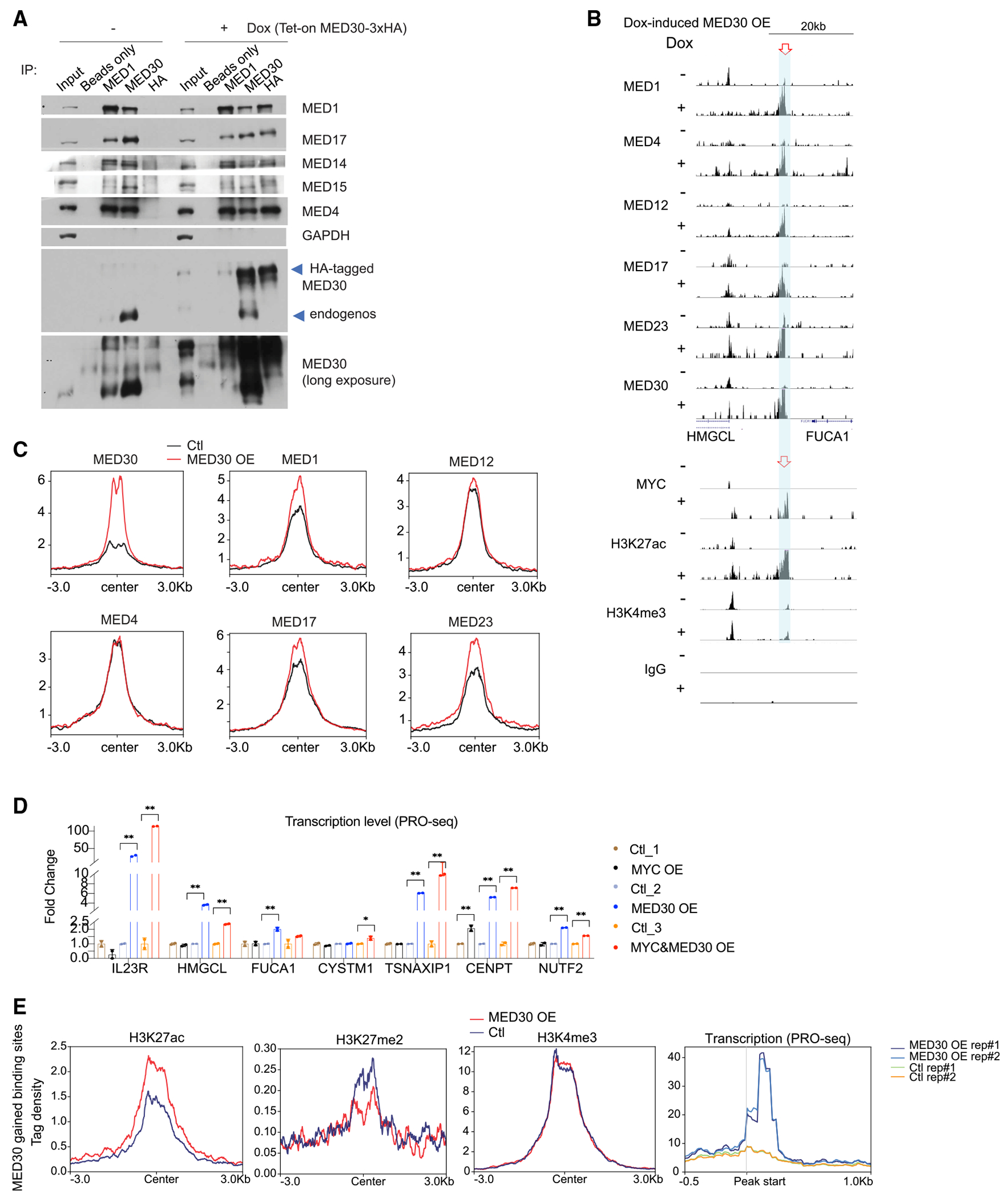
MED30 license mediator complex and MYC to bind to genomic sites at both basal and OE condition (A) CoIP experiments in Tet-on MED30-3xHA Mia PaCa-2 cells. (B) Genome browser example of indicated mediator subunits and other factors in gained enhancer upon MED30 OE (+Dox), by CUT&Tag experiments in Mia PaCa-2 cells. (C) MED1, MED4, MED12, MED17, MED23, and MED30 tag density profile on MED30 OE gained peaks Mia PaCa-2 cells (CUT&Tag experiment). (D) Transcription level (PRO-seq) changes of the exemplified genes with gained mediator binding peaks nearby. Data are presented as mean ± SD. **p < 0.01; two-tailed Student’s *t* test. (E) Tag density profile of indicated histone marks and transcription level (PRO-seq) on MED30 gained sites.

Overexpressed MED30 also interacts with other subunits of the mediator complex (Figure 4A). To determine whether MED30 alone or the broader mediator complex mediates the transcriptional activation observed upon MED30 OE, we examined the recruitment of additional mediator subunits. We first performed CUT&Tag for MED1 in Tet-on MED30-overexpressing Mia PaCa-2 cells, with or without doxycycline induction for 2 days. The results revealed a strong correlation between MED1 and MED30 binding: sites that gained MED30 also gained MED1, while sites that lost MED30 similarly exhibited reduced MED1 binding (Figures S4A and S4B). This effect was specific to MED30 OE and not due to doxycycline treatment itself, as no such changes were observed in a Tet-on shMED30 knockdown cell line, with or without doxycycline (Figure S4C). To further validate and extend these findings, we performed CUT&Tag for additional mediator subunits MED4, MED12, MED17, and MED23 alongside MED1 and MED30, in Mia PaCa-2 cells overexpressing HA-tagged MED30 (Figure 4B). Genome-wide analysis confirmed that MED30 binding gains were highly reproducible across independent experiments. Notably, other mediator components from distinct modules also showed increased binding at MED30-gained sites (Figure 4C). The most significantly upregulated MED30 peaks exhibited strong co-occupancy by all tested mediator subunits (Figure S5A). Furthermore, genes located adjacent to these MED30-gained peaks showed significantly elevated expression (Figure 4D), supporting the conclusion that MED30 OE promotes assembly of the mediator complex at newly engaged regulatory regions, thereby driving transcriptional activation.

### MED30 OE is associated with activation new enhancer regions

We next investigated whether the histone landscape is altered in parallel with changes in mediator and MYC binding upon MED30 OE. To this end, we performed CUT&Tag in doxycycline-induced MED30-overexpressing Mia PaCa-2 cells, assessing the active enhancer mark H3K27ac, the repressive/poised enhancer mark H3K27me2, and the active promoter mark H3K4me3. Genome browser tracks confirmed the expected distribution: H3K4me3 was localized to gene promoters, H3K27ac marked both promoters and intergenic regions, and H3K27me2 was broadly distributed across the genome but excluded from active regions (Figure S5B). Mapping MED30-gained and -lost binding sites to these histone modifications revealed that gained sites were associated with increased H3K27ac and decreased H3K27me2, while H3K4me3 remained largely unchanged (Figures 4 and S5C). In contrast, MED30-lost sites showed a decrease in H3K27ac but no significant changes in H3K27me2 or H3K4me3. The relatively low levels of H3K27ac and H3K4me3 at the lost sites suggest that these regions were not associated with active enhancers or promoters (Figure S5C). Thes newly formed H3K27ac peaks represent only a small subset of genome-wide enhancers (Figure S5D), which is consistent with the relatively modest number of genes transcriptionally regulated by MED30 OE. Collectively, these results indicate that MED30 OE promotes the establishment of active enhancers by increasing H3K27ac deposition at gained binding sites, which is accompanied by elevated enhancer RNA (eRNA) production and transcriptional activation of nearby target genes.

### MED30 OE promotes pancreatic cancer cell growth *in vitro* and *in vivo*

**Figure 5.**
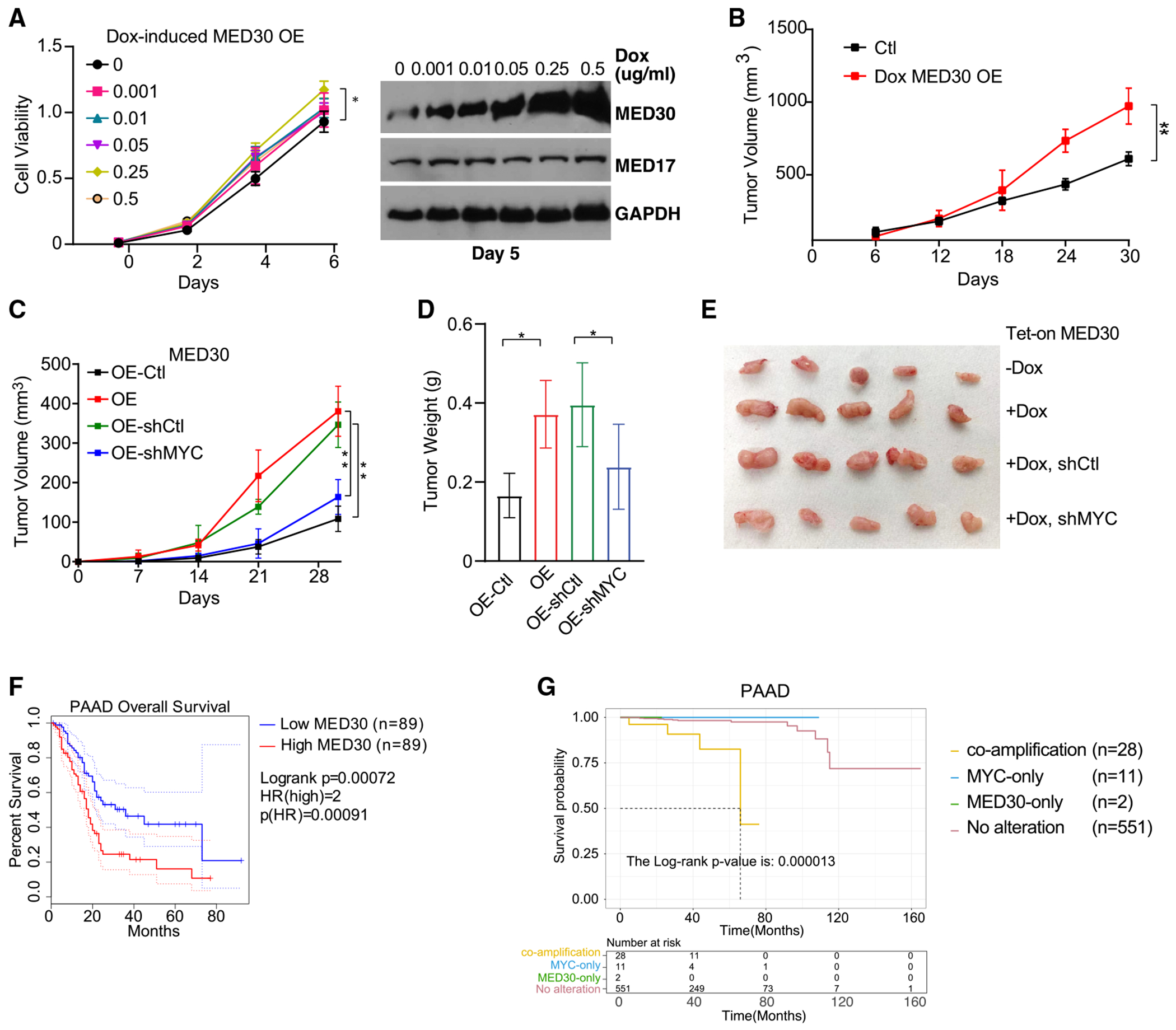
MED30 promotes pancreatic cancer cell and tumor growth *in vitro* and *in vivo* (A) Cell proliferation assay for MED30-overexpressed Mia PaCa-2 cells, in indicated concentration of Dox (μg/mL). *p < 0.05, two-tailed Student’s *t* test. Right: western blot showing the protein level of gradient dox-induced MED30 OE. (B) Tumor growth curve of Mia PaCa-2 with Dox-induced MED30 OE (control: no Dox) in nude mice xenograft (*n* = 5 for each group). **p < 0.01, log rank test. (C) Tumor growth curve of Mia PaCa-2 with Dox-induced MED30 OE (control: no Dox), and shControl (non-target shRNA) or shMYC in nude mice xenograft (*n* = 5 for each group). *p* values calculated using log rank test, *p < 0.05, **p < 0.01. (D) Tumor weight and for each group of (C). *p* values calculated using two-tailed unpaired Student’s *t* test. *p < 0.05, **p < 0.01. Data are presented as mean ± SD for (A–D) (E) Tumor image at the day30 for (C). (F) Survival curve of high and low MED30 expression level in pancreatic cancer patients (data from GEPIA). (G) Overall survival curve for no alteration or amplification of MED30-only, MYC-only, or MED30/MYC-both in pancreatic cancer patients (data from TCGA).

Given the frequent amplification of *MED30* in cancer, we investigated its functional contribution to tumor growth. We first examined the effects of graded *MED30* OE on the proliferation of Mia PaCa-2 cells. Doxycycline (Dox)-induced *MED30* OE modestly enhanced cell growth *in vitro* (Figure 5A). Since tumor behavior can differ significantly between *in vitro* and *in vivo* contexts, we next evaluated the impact of *MED30* OE in a xenograft model. In nude mice, Dox-induced *MED30* OE significantly promoted Mia PaCa-2 tumor growth (Figure 5B). To determine whether the tumor-promoting effects of MED30 were dependent on MYC, we generated Mia PaCa-2 cells with Dox-inducible MED30 OE and stably expressed either a non-targeting control shRNA (shCtl) or MYC-targeting shRNA (shMYC). While MED30 OE with shCtl preserved the tumor growth-promoting effect, *MYC* knockdown significantly attenuated this effect, indicating that MED30’s oncogenic activity is MYC-dependent (Figures 5C–5E). To further explore the consequences of MED30 perturbation, we performed a series of cell-based assays following *MED30* knockdown. Loss of MED30 increased DNA damage, as indicated by immunostaining for the double-strand break marker γH2AX (Figure S6A), and elevated apoptosis, as measured by Annexin V staining (Figure S6B). These findings underscore MED30’s role in supporting pancreatic cancer cell survival and proliferation.

To assess the clinical relevance, we analyzed MED30 expression and patient outcomes in pancreatic adenocarcinoma (PAAD). High MED30 expression was significantly associated with poor overall survival (Figure 5F). We also evaluated patient survival in relation to gene amplification status using the ATCC database. Amplification of either *MYC*- or *MED30*-only gene was associated with worse survival outcomes, with the strongest effect observed in co-amplified cases (Figure 5G).

### MED30 and MYC co-amplification functions across multiple cancer types

**Figure 6.**
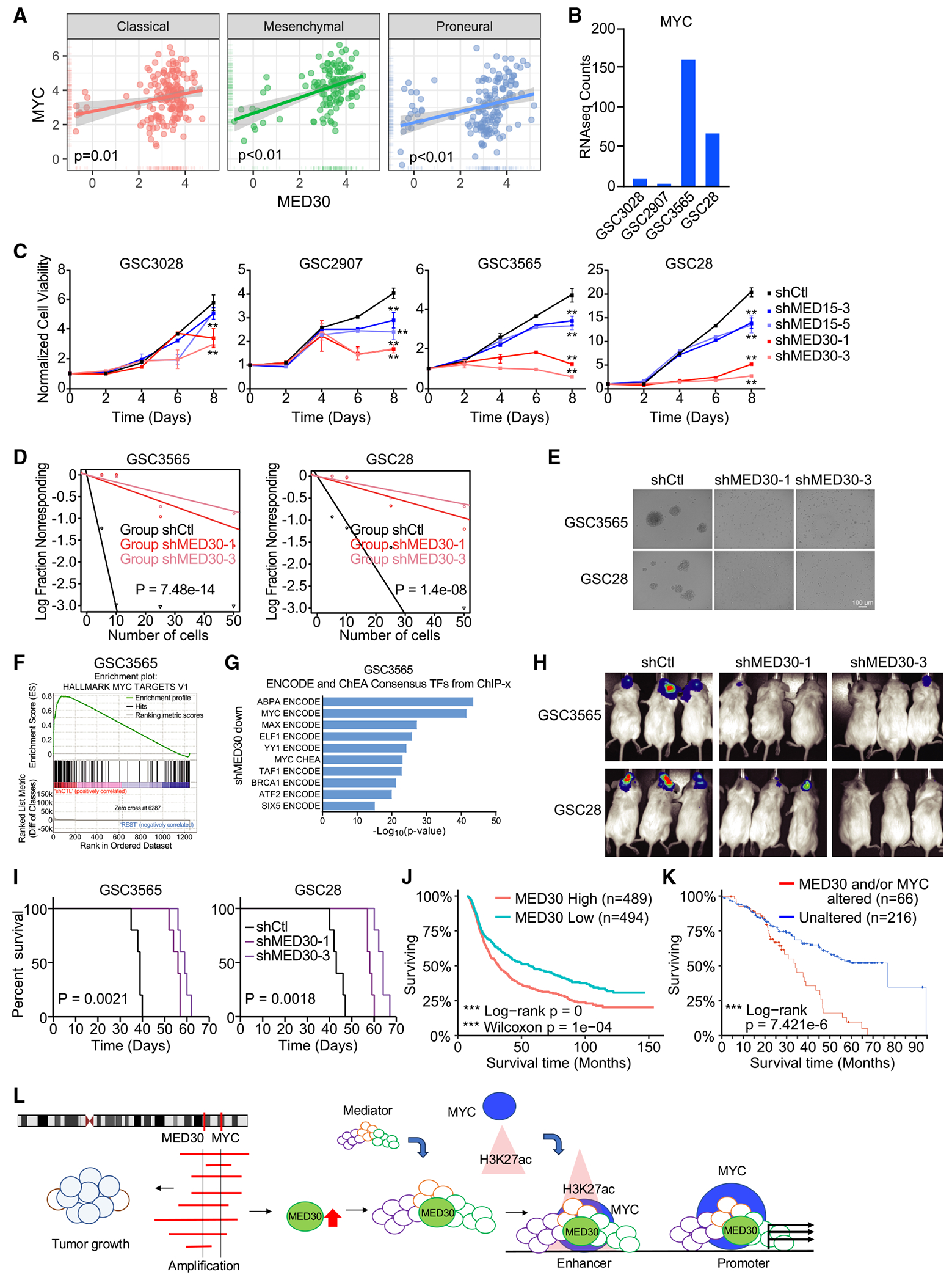
MED30 promotes brain cancer cell and tumor growth *in vitro* and *in vivo* (A) Pearson correlation analysis of MED30 and MYC gene expression level in brain tumors using CGGA dataset, separated by subtype (http://gliovis.bioinfo.cnio.es/). (B) Expression level of MYC in different glioblastoma cells (GSC3028, GSC2907, GSC3565, and GSC28), revealed by RNA-seq. (C) Cell proliferation assay of shMED30 or shMED15 knockdown in indicated glioblastoma cells. **p < 0.01, two-tailed Student’s *t* test. (D) Sphere formation using an extreme limiting dilution assay (ELDA) was performed with 3,565 and 28 GSCs expressing control shRNA (shCtl) or one of two independent, non-overlapping shRNAs targeting MED30. (E) Images in ELDA assay (scale bars, 100 μm). (F) GSEA analysis of MED30 knockdown RNA-seq regulated gene in GSC3565 cells. (G) ENCODE and ChEA consensus TFs from ChIP-X analysis using online “Enrichr” program. (H) Bioluminescence imaging of nude mice carrying GSC3565 or MES28 with or without shMED30 knockdown glioblastoma xenograft labeled by luciferase. (I) Survival curve of nude mice carrying GSC3565 or MES28 with or without shMED30 knockdown glioblastoma xenograft (*n* = 5 for each group, *p < 0.05, **p < 0.01 log rank test). (J) Overall survival analysis of MED30 gene expression level with brain tumor patient survival rate in CGGA dataset (http://gliovis.bioinfo.cnio.es/). Data contain all histology and subtype samples. (K) Overall survival analysis of pan-cancer patients with or without altered MED30 and/or CNVs (data from cBioportal > ICGC/TCGA, Nature 2020). (L) Working model of MED30 and MYC co-amplification in tumorigenesis.

Since MED30 gene amplification occurs across nearly all cancer types, we investigated its functional role in another aggressive cancer—brain cancer. Consistent with previous findings of co-amplification, MED30 and MYC expression are significantly correlated across all major brain tumor subtypes: classical, mesenchymal, and proneural (Figure 6A) as well as in most histological types (Figure S7A). Glioblastoma (GBM), a major subtype of brain cancer, contains self-renewing, tumor-initiating glioblastoma stem cells (GSCs).^39^ Analysis of TCGA GBM RNA-seq data revealed a positive correlation between MED30 expression and tumor grade (Figure S7B). To assess whether GSC proliferation and stemness are dependent on MED30, and how this correlates with MYC expression, we used a panel of patient-derived GSC lines (GSC2907, GSC3028, GSC3565, and GSC28) with varying levels of MYC (Figure 6B). We previously demonstrated that GSCs rely on MYC for proliferation and self-renewal.^40^ As a control, we used MED15, a non-essential mediator subunit whose knockdown does not affect levels of other subunits.

In proliferation assays, MED30 knockdown markedly inhibited GSC growth, much more so than MED15 knockdown (Figure 6C), despite the knockdown efficacy was similar (Figure S6C). This effect was especially pronounced in high-MYC GSCs (GSC3565 and GSC28) compared to low-MYC lines (GSC2907 and GSC3028), suggesting that MYC-high tumors are more dependent on MED30 (Figure 6C). Furthermore, neurosphere formation, a readout of stemness, was significantly reduced upon *MED30* knockdown in GSC3565 and GSC28 cells (Figures 6D and 6E).

To investigate transcriptional changes, we performed RNA-seq in GSC3565 cells transduced with two different shRNAs targeting MED30. Both shRNAs achieved high knockdown efficiency (~90%) and showed consistent transcriptomic profiles (Figure S7C). After 2 days of knockdown, ~2,300 genes were downregulated and ~2,400 upregulated across both shRNAs (Figure S7D). Downregulated genes were enriched for essential cellular functions, such as ribosomal proteins, electron transport, and oxidative phosphorylation, indicating widespread transcriptional disruption (Figure S7E). GSEA analysis revealed strong enrichment of MYC target genes among the downregulated transcripts (Figure 6F), and MYC was among the top TFs identified in motif enrichment analysis (Figure 6G), consistent with findings in Mia PaCa-2 cells.

To assess *in vivo* relevance, we orthotopically implanted GSC3565 and GSC28 cells expressing shCtl or shMED30 into the brains of nude mice. MED30 knockdown significantly impaired tumor growth and extended survival in both models (Figures 6H and 6I). Analysis of a broad cohort of adult brain tumor patients, spanning all tumor types (primary, recurrent, and secondary) and histologies (e.g., oligodendroglioma, astrocytoma, and GBM), showed that high MED30 expression correlates with poor survival (Figure 6J). Given the ubiquitous expression of MED30 and MYC across tissues (Figure S7F), we propose a mechanism that may represent a broadly relevant mechanism across cancers. Indeed, pan-cancer analysis using ICGC/TCGA data via cBioPortal confirmed that amplification of MED30 and/or MYC is associated with reduced overall survival across multiple tumor types (Figure 6K). In summary, the functional synergy between MED30 and MYC described here is likely a common oncogenic mechanism across diverse cancers. Collectively, our results suggest a model in which MED30 amplification drives the gain of new enhancers, leading to the recruitment of other mediator complex subunits and TFs such as MYC. This enhancer activation reprograms the transcriptome toward a state that promotes cell proliferation and tumor growth, ultimately correlating with poor patient outcomes (Figure 6L).

## DISCUSSION

With the increasing realization that many gene enhancers harbor low affinity binding sites for their TFs,^41^ it becomes important to investigate whether the activity of many oncogenic TFs is constrained because low-affinity sites often remaining unbound under physiological conditions. In this manuscript, we identify a mechanism by which amplification of a coactivator, the MED30 subunit of the mediator complex promotes aberrant MYC binding to its low-affinity enhancers and promoters, with deleterious prognostic outcomes. Thus, selective amplification or OE of MED30 alone is sufficient to empower MYC to bind and activate previously unoccupied or weekly bound sites in cancer cells, driving expression of genes critical for tumor progression. Despite decades of research, the mechanisms by which MYC exerts its oncogenic functions-particularly in relation to the levels of the mediator complex—remain incompletely understood. This reveals an underappreciated aspect of MYC’s transcriptional and oncogenic program. Our findings highlight the quantitative role of mediator complex abundance in selectively regulating oncogenic gene activation programs.

Located in close genomic proximity on chromosome 8 (MYC at 8q24.21 and MED30 at 8q24.11), MYC and MED30 are frequently co-amplified across multiple cancer types. We identify a previously unrecognized mechanism by which the MED30-containing mediator complex facilitates MYC recruitment to previously unoccupied regulatory elements harboring low affinity MYC binding sites, thereby driving additional oncogenic transcriptional programs. This functional interplay links two recurrent genetic alterations and underscores the potential biological significance of co-amplification events. More broadly, these findings suggest that co-occurring genomic alterations—particularly involving genes not traditionally viewed as oncogenic drivers—including coactivators—may play critical, yet overlooked, roles in enhancing the activity of well-established oncogenes. This example of the role of oncogenic TF-associated factors to license binding to additional regulatory elements as a mechanism increasing pathologic transcriptional programs in cancer to likely to actions of many oncogenes and warrants further investigation into the cooperative contributions of co-amplified genes to tumorigenesis.

### A component of the mediator complex is critical for MYC recruitment and function

The epidemiological link between amplification of the mediator complex subunit MED30 and cancer provides key insights into both the molecular mechanisms by which MED30 OE drives tumorigenesis and the essential role of MED30 in mediator function. Importantly, MED30 OE facilitates the recruitment of MYC to previously unoccupied or weakly bound regulatory elements, expanding the range of MYC-bound enhancers and promoters. Accordingly, knockdown of MYC abolishes the MED30-induced transcriptional activation program in both cell culture and *in vivo* models. Conversely, MED30 depletion leads to a marked reduction in MYC genomic binding. As a core component of the mediator complex, MED30 is critical for stabilizing other mediator subunits, supporting overall complex integrity and transcriptional regulation.^42^

### MED30 is one of the highest genetic amplified genes in pan-cancer and is a potential therapeutic target

Previous studies implicate MED30 in multiple cancers. It promotes proliferation, migration, and invasion.^43^ In GBM, its expression is induced by hypoxia and nutrient stress via HIF1α/p53, enhancing proliferation and temozolomide sensitivity.^44^ MED30 OE has been linked to poor prognosis in renal cell carcinoma, where its knockdown suppresses tumor cell growth and motility.^45^ In oral squamous cell carcinoma, MED30 was associated with a recurrence-related gene signature and oxidative phosphorylation, correlating with adverse survival outcomes.^46^ Tens of thousands of patient sample profiling data accumulated in database showed that MED30 gene amplification is present at high level in widespread of cancer types. In cancer of breast, liver, ovary, uvea, or uterus, the homozygous amplification rate is around or above 20%. This variation frequency on average is lower than TP53 mutation, similar to that of MYC and higher than other well-known oncogenes, for example, PTEN, KRAS, and PIK3CA, suggesting that MED30 may serve as a biomarker for cancer subtypes that are potentially regulated by MED30 targeting.

MYC binds as a homodimer or heterodimer to its DNA binding elements, referred to an E-boxes,^47–49^ and the absence of MAX augments binding despite increasing oncogenesis in B cells.^50^ Our study reveals that MYC homo- or hetero-dimer binding to the cognate E-box sites on promoters and enhancers is increased based of the ability of MED30 to interact with MYC. Establishing the interactions of MED30 and MYC establish the role of MED30 in the oncogenic actions of MYC.

Thus, in cases of isolated MED30 or MYC amplification, or even in the absence of their amplification, MED30 is a critical determinant of the program by which MYC activates growth of multiple tumor types and implies the potential therapeutic benefit of inhibition of MED30 levels or the interaction with other mediator subunits. Given the high number of cancer patients harboring MYC and/or MED30 amplification, targeting MED30 might have wide application in antagonizing cancer progression. Therefore, increasing levels of a specific coactivator, in this case MED30, caused a limited cohort of regulatory elements to be activated, requiring binding of a specific DNA-binding TF, MYC. This suggests that limited number of TFs will be affected by such coactivator OE, in this case, the limited activation program increases the risk of cancer progression.

### Limitations of the study

While our findings demonstrate MED30 OE promotes cancer cell growth and provide a mechanistic basis for how MED30 amplification licenses MYC binding at low-affinity enhancers, several limitations warrant consideration. First, our study mechanistic studies are primarily performed in Mia PaCa-2 cells, while the co-amplification of MYC and MED30 is observed across diverse cancer types, the specific oncogenic programs activated may vary in different context. We cannot exclude the possibility that other transcription factors also collaborate with MED30 to drive tumor progression in different cells. Second, from biochemical perspective, the extent to which MED30 is required for mediator complex stability, and whether other subunits exert similar functions remain to be fully defined. We are exploring these questions. Finally, our therapeutic implications are based on genetic knockdown models; the development and testing of small-molecule inhibitors targeting the MED30-associated Mediator assembly, or MED30-MYC association will be necessary to establish the clinical feasibility.

## RESOURCE AVAILABILITY

### Lead contact

Further information and requests for resources and reagents should be directed to and will be fulfilled by the lead contact, Michael G. Rosenfeld (mrosenfeld@ucsd.edu).

### Materials availability

This study did not generate new unique reagents. We are glad to share with reasonable compensation by the requestor for its processing and shipping.

## STAR★METHODS

### EXPERIMENTAL MODEL AND STUDY PARTICIPANT DETAILS

#### Human cell lines and culture

Mia PaCa-2, HEK293T cells were obtained from ATCC were cultured in DMEM (GIBCO_ #10566) media supplemented with 10% FBS. Cell lines were maintained using standard tissue culture techniques. Glioblastoma tissues that were used for isolation of GSCs were obtained from excess surgical resection samples from patients at the Case Western Reserve University after review by neuropathology with appropriate consent and in accordance with an IRB-approved protocol (090401). GSCs were cultured in Neurobasal media (Invitrogen) supplemented with B27 without vitamin A (Invitrogen), EGF and bFGF (20 ng/mL each; R&D Systems), sodium pyruvate, and glutamax. All cells were cultured in a 5% CO2 humidified incubator at 37°C. Mycoplasma negativity was ensured routinely.

#### Animal models

NSG (NOD.Cg-Prkdcscid Il2rgtm1Wjl/SzJ) mice were purchased from the Jackson Laboratory, Bar Harbor, ME, USA. All animal experiments were approved by the Institutional Animal Care and Use Committee of UCSD.

### METHOD DETAILS

#### Construct, lentivirus packaging, and stable cell line generation

pLKO lentiviral shRNA constructs and control shRNA constructs were purchased from Sigma (See Table S2). Teton shRNA constructs were homemade by annealing the shRNA sequence to pLKO.1 vector (addgene#326721). Knockdown experiments with lentivirus shRNAs were conducted according to the standard lentivirus package and transduction protocols from Addgene or Sigma. For cDNA overexpression experiments, MED30 or Myc cDNA was cloned into pTEPRT vector, and For ChIP experiment, the constructs were integrated with C-terminal or N-terminal 3XHA tag. This vector is doxycycline-inducible all-in-one construct with improved tetracycline controlled transactivator (TetR) and target gene expression driven by tandem Tet operators. The constructs carry puromycin resistant gene for positive selection. For the induction of target gene expression, titrated doxycycline was supplemented into the culture medium to achieve an expression of near endogenous level. pLKO-based lentiviral shRNA plasmids were co-transfected with packaging plasmids (psPAX2 and pMD2.G) into 293 T cells. Lentiviruses were harvested, concentrated, and used for cell infection. Stable knockdown Mia PaCa-2 cells were selected with 0.4 mg/mL puromycin and collected for experiments within 5 days. For doxycycline induced knockdown or overexpression, the concentration of doxycycline was 0.5 μg/mL unless specified.

#### qPCR, data analysis, and statistical analysis

For qRT-PCR experiments, the Mia PaCa-2 or glioblastoma cells with indicated shRNA knockdown or gene overeverexpression were collected RNA was isolated using RNA extraction kit (Zymo Research) or RNeasy column (QIAGEN). Total RNA then was reverse-transcribed using SuperScript III Reverse Transcriptase(Life Technologies) or following manufacturer’s instructions. qPCRs were performed in MX3000P (Stratagene) using 2× qPCR master mix from Affymetrix or Bio-Rad. Relative quantities (RQ) of gene expression levels were normalized to GAPDH unless specified. A list of primers used for qPCR is provided (Key Resources Table).

For all qRT-PCRs, experiments were performed with at least three independent biological replicates and three technical replicates for each reaction. Results are reported as mean ± SD of a representative batch Data were analyzed and statistics were performed using unpaired two-tailed Student’s t tests. Significant differences between two groups were noted by asterisks (*p < 0.05, **p < 0.01, ***p < 0.001).

#### RNA-seq and analysis

To examine the transcriptome influence of depleting difference mediator subunit, shRNAs target MED1,MED20, MED30 or non-target Ctrl were packaged into lenti-virus and infected into Mia PaCa-2 cells, and total RNAs were extracted two days post infection. MED30 wild-type or mutant overexpression comparing to knockdown RNA seq in Mia PaCa-2 was performed with 0.5 μg/mL doxycycline-induced teton shRNA or cDNA overexpression, and total RNAs were extracted two days post Dox induction. All shRNAs were packaged in lentivirus and infected into cells. RNA was extracted by Trizol (Life Technologies) RNA concentration and quality was quantified by Qubit and Agilent 2100 Bioanalyzer system, respectively. Libraries were generated using illumina PolyA RNA library kit and sequenced by HiSeq4000 with single-end reads or NovaSeq 6000 with 100 bp paired-end reads.

Following sequencing, quality control with FastQC and trimming of adaptor sequence and poly-A tails with Trim Galore, reads were mapped and assigned to the human transcripts using HISAT2. The counts file was generated by “featureCounts” on Galaxy (https://usegalaxy.org/) with build-in genome hg38. Differential expression gene (DEG) analysis was performed using the DESeq2 package in Galaxy. Perimeters for different expression cutoff were indicated in figure legend. Functional analysis of differential expressed genes were performed on Enrichr (https://maayanlab.cloud/Enrichr/).

#### Chromatin immunoprecipitation (ChIP)

5 million cells were dual cross-linked with 2 mM EGS (ethylene glycol bis(succinimidyl succinate)) for 20min at room temperature and followed by 1% formaldehyde for 10 min for Mediator’s ChIP. The cross-linking was then quenched with 0.125 M glycine for 5 min. Chromatin was fragmented in lysis buffer (20 mM Tris-HCl pH 8.0, 150 mM NaCl, 2 mM EDTA, 1% SDS, 1× protease inhibitor cocktail) using a Bioruptor to get 200-500bp fragments. Subsequently, the soluble chromatin was diluted 10-fold by dilution butter (20 mM Tris-HCl pH 7.8, 150 mM NaCl, 2 mM EDTA, 1% Triton X-100) and incubated with 1-3 ug antibodies at 4°C overnight. The next day 10-20 ul Protein G Dynabeads (Life Technologies) were added into the reaction and incubate for 3 h with rotation at 4°C. Then beads captured complex were washed with TSE I (20 mM Tris-HCl pH 7.8, 150 mM NaCl, 2 mM EDTA, 1% Triton X-100) for 10 min twice TSE II (20 mM Tris-HCl pH 7.8, 450 mM NaCl, 2 mM EDTA, 1% Triton X-100) for 10min, and TE (10mM Tris-HCl pH 8.0, 1 mM EDTA) twice. After washing, the protein-DNA complexes were eluted with elution buffer (TE +150 mM NaCl, 1% SDS) and de-crosslinked overnight at 65°C. and DNA was purified with QIAquick PCR Purification Kit (Qiagen, # 28104).

#### CUT&Tag-seq and analysis

Bench top CUT&Tag version 3 was performed as previously described,^51^ with modifications. Specifically, cells were seeded in 24-well plate at 30–50% confluency, and treated with indicated doxycycline concentration for two days to allow the induction of shRNAs or cDNA plasmid, and the attachment to the well’s bottom. On the day of experiment, cells in culture media were lightly fixed (0.1% formaldehyde for 2 min) for non-histone epitopes or without fixation for histone marks, and quenched glycine of >twice molar concentration of formaldehyde. Next, the cells were washed with PEX buffer (PBS, 2mM EDTA, 0.1% Triton X-100) for 5min thrice, in order to complete remove Mg2+ and permeabilize the cell. Then the cells were blocked by antibody buffer (0.1%BSA, 2mMEDTA) for 10-30min at room temperature, and subjected to primary antibody incubation overnight at 4°C. The antibodies were used as 1:1000 dilution in antibody buffer if not specified. The next day the primary antibody was removed and wash with wash buffer (20mMTrisCl pH7.4, 150mMNaCl, 0.05%Triton X-100) for 5min, and then incubated with secondary antibody 1:2000 diluted in wash buffer for 1 h. The secondary antibody contains fluorophore (Alexa Fluor) so the signal can be checked under microscope. After secondary antibody incubation, cells were washed three times with wash buffer, and then add adapter loaded pAG-Tn5 (purified in-house^52^) in Dig-300 buffer (20mMTrisCl pH7.4, 300mMNaCl, 0.05%Triton X-100) for 1 h, followed by three time wash in Dig-300 buffer. Then cells were subjected to tagmentation buffer (10mMMgCl_2_ in Dig-300 buffer) at 37°C for 1-3 h, and terminated and de-crosslinked by adding 7.5 μl 0.5MEDTA, 8 μl 10%SDS, 0.25ul 20mg/mL proteinase K per 150 μl tagmentation buffer in one well and incubated at 65°C for 30 min, and transferred into Eppendorf tube to avoid evaporation and continue de-crosslinking overnight, followed by DNA purification with DNA purification Kit (Qiagen). The DNA was eluted and utilized as template for PCR reaction with i7 and i5 dual-index for library construction and sequenced on the NovaSeq 6000 with 100 bp paired-end reads at the UCSD IGM Genomics Center. Because the sequencing is deep, the signal is not sparse. The adapters were trimmed by Trim Galore and aligned to the hg38 assembly by Bowtie2. bigwig files were generated using the DeepTools “BamCoverage” script integrated in Galaxy server by normalizing to CPM (counts per million), and uploaded to UCSC genome browser for visualization. DeepTools “computeMatrix”, “plotHeatmap”, and “plotProfile” functions were used to generate heatmaps and profile plots. Peaks were identified by MACS2. Peak file was uploaded to UCSC genome browser aligned with BamCoverage (bigwig) to check the reliability of peak calling. Motif analysis was performed using HOMER software package (http://homer.ucsd.edu/homer/motif). Annotation of peaks was performed using R package “ChIPseeker” with “hg38.refseq.gtf.gz” from UCSC Genome Browser as reference.

#### PRO-seq and analysis

PRO-seq was performed in doxycycline (Dox) inducible MED30, MYC-3xHA or MED30&MYC-3xHA double integrated Mia PaCa-2 cell line with or without 0.5μg/mL Dox treatment for 1 day. Samples with Dox treatment were labeled as OE (overexpression). Each condition has two replicates. Precision run-on sequencing (PRO-seq) experiments were performed as previously described.^53^ For nuclei isolation, cells were incubated with swelling buffer (10 mM Tris-Cl pH 7.5, 2 mM MgCl_2_ and 3 mM CaCl_2_) for 5 min on ice and then incubated with lysis buffer (swelling buffer with 0.5% NP-40 and 10% glycerol) for 5 min on ice, before being re-suspended in 100 μL of freezing buffer (50 mM Tris-Cl pH 8.0, 40% glycerol, 5 mM MgCl_2_ and 0.1 mM EDTA). For the run-on assay, an equal volume of reaction buffer (10 mM Tris-Cl pH 8.0, 5 mM MgCl2, 300 mM KCl, 1 mM dithiothreitol, 20 units of SUPERase·In, and 500 μM ATP, GTP, bio-UTP and bio-CTP) was added into each sample before incubation at 30°C for 5 min. The nuclear run-on RNA was then extracted with TRIzol LS reagent (10296010, Invitrogen) and subjected to hydrolysis, buffer exchange and purification by streptavidin beads (88816, Thermo Fisher). Purified RNA was treated with PNK before being used for complementary DNA synthesis by using the NEBNext Multiplex Small RNA Library Prep Set for Illumina Kit (E7300S, NEB). Obtained complementary DNA template was amplified by PCR using the Phusion High-Fidelity enzyme (M0530L, NEB) for deep sequencing.

The sequencing reads were trimed by Trim Galore. The adapter sequence to be trimed was selected as “automatic detection” for the first round and the “Illumina small adapters” for the second round (Galaxy Version 0.6.7+galaxy0). Then the reads were mapped to hg38 Refseq database by using Bowtie2 and counted over the entire gene body (transcript) on the sense strand with respect to the gene orientation by using Featurecounts (Galaxy Version 2.0.3+galaxy1). Deseq2 (Galaxy Version 2.11.40.8+galaxy0) was then used to compute the significance of the differential gene expression.

#### Immunoprecipitation (IP) and western blot

For MED30 and MYC co-IP, 5 million cells were rinsed with ice-cold PBS and then cross-linked with 1mM EGS at room temperature for 20min, and quenched 0.125 M glycine. Then lysed in lysis buffer (150 mM NaCl, 20 mM Tris-Cl pH7.4, 1% Triton X-100) with protease inhibitors (Roche, #11873580001), rotating at 4°C for 30 min and centrifuged at 12,000 rpm for 10 min. For co-IP among Mediator subunits, cross-link was omitted. After that, 1 μg primary antibodies were added into the supernatant cell lysate and incubated overnight 4°C with rotation. Then 10 μl (for 1 μg antibody) Protein A/G magnetic beads (Pierce, #88803) were added and incubated with rotation for 3 h at 4°C. Immunoprecipitants were washed four times with lysis buffer and were denatured by SDS-PAGE sample buffer and boiled for 5 min. Sample were followed by immunoblot analysis. Immunoblot was performed per a general western-blot protocol (Abcam). antibodies are listed in KEY RESOURCES TABLE.

#### Cell proliferation assays

Proliferation assay for Mia PaCa-2 cells was performed in two ways: one is violet staining; the other is measured by MTT (Sigma) reagent. 3000–6000 cells per well with indicated treatment were seeded in 96-well plate with 6 replicates. MTT was dissolved in PBS at 5mg/mL (10× working solution) and added 10 μl to 100 μl culture medium and incubated for 3 h in the cell culture incubator. After incubation, supernatant was discarded and the plate was scanned for taking picture. Then use 100 μl DMSO to dissolve the dark blue product generated in the cell, and 540nm absorbance was measured after a few minutes’ shaking. Proliferation assay for glioblastoma cells: Cell proliferation experiments were performed by plating cells at a density of 1000 cells/well in a 96-well plate with three to six replicate wells. CellTiter-Glo (Promega) was used to measure cell viability at day 0,1,3,5 and 7. All data were normalized to day 0.

#### Immunofluorescence for DNA damage detection

Mia PaCa-2 cells with Tet-on shMED30 were grow on coverslip and 0.5 μg/mL doxycycline or vesicle ethanol was added to culture medium for two days before immunostaining. Then the cells on coverslip were washed by PBS twice, fixed with 4% paraformaldehyde for 15min and permeabilized by 0.1% Triton X-100 in PBS for 20min at room temperature. Next, the cells were blocked by 3% BSA and sequentially incubated with the primary antibody for 1.5hr and the secondary antibody for 1hr at room temperature. Last, coverslips were mounted by mounting medium with DAPI (Vector Laboratories) and sealed with nail polish before subjected to microscopy.

#### Apoptosis assay

Teton shMED30 Mia PaCa-2 cells were treated with or without 0.5 μg/mL doxycycline for four days and then subjected to analyze by FITC Annexin V Apoptosis Detection Kit I (BD Pharmingen) following manufacturer’s protocol.

#### Neurosphere formation assay

Neurosphere formation was measured by *in vitro* limiting dilution assay, as previously reported.^54^ Briefly, decreasing numbers of cells per well (50, 20, 10, 5 and 1) were plated into 96-well plates. The presence and number of neurospheres in each well were recorded seven days after plating. Extreme limiting dilution analysis (ELDA) was performed using software available at http://bioinf.wehi.edu.au/software/elda.

#### Tumor xenograft models

For intracranial tumor xenograft, GSCs (10^4^/mouse, 20 μL of Neurobasal media with no Matrigel) were intracranially implanted into NSG mice (NOD.Cg-Prkdcscid Il2rgtm1Wjl/SzJ, The Jackson Laboratory, Bar Harbor, ME, USA). Mouse brains implanted with GSCs which were labeled with firefly luciferase were monitored by the bioluminescent imaging at the 4th week post injection. Animals were treated with D-Luciferin (120mg/kg, Biosynth Carbosynth, no. L-8220) intraperitoneally and anesthetized with isoflurane for the imaging analysis. The bioluminescent images were captured by an IVIS imaging system (Spectrum CT, PerkinElmer).

For Mia PaCa-2 MED30 overexpression xenograft, doxycycline-induced MED30 construct was integrated into Mia PaCa-2 cells to establish stable cell line. Lentivirus shControl or shMyc was infected into cells two days before injection. For Mia PaCa-2 shMED30 knockdown xenograft, cells were infected with lentivirus shControl or two shMED30 respectively two days before injection. Then NSG mice (NOD.Cg-Prkdcscid Il2rgtm1Wjl/SzJ, The Jackson Laboratory, Bar Harbor, ME, USA) were implanted with 10^6^ cells by subcutaneous injection, and random divide into Dox and non-Dox group (*n*=5 each). Dox group was supplied with 1mg/mL doxycycline in drinking water in three days post injection. Tumor dimensions were measured once when tumors were palpable. Tumor volumes were monitored twice a week till calculated using the equation (length^2^ × width)/2.

## Supplementary Material

1

2

3

Supplementary data related to this article can be found online at https://doi.org/10.1016/j.celrep.2026.117498.

## Figures and Tables

**Table:** KEY RESOURCES TABLE

REAGENT or RESOURCE	SOURCE	IDENTIFIER
Antibodies		
MED1	Bethyl	A300-793 A
MED7	Santa Cruz	E-4
MED8	Santa Cruz	A-5
MED12	Bethyl	A300-774 A
MED13	Bethyl	A301-278A-1
MED14/CRSP2/DRIP150	Bethyl	A301-044 A-T
MED15	Bethyl	A302-423 A
MED16	Bethyl	A303-668 A-T
MED18	Bethyl	A300-777 A-T
MED22	Santa Cruz	D-10
MED23/CRSP3	Bethyl	A300-425 A-T
MED24/TRP100	Bethyl	A301-472
MED17	invitrogen	PA5-30314
HA-Tag	Cell Signaling	#3724
c-Myc	Cell Signaling	#9402
c-Myc	Cell Signaling	#18583
GAPDH	Santa Cruz	FL-335
MED30	Dr. Thomas G. Boyer, University of Texas Health Science Center at San Antonio	homemade
MED4	Dr. Thomas G. Boyer, University of Texas Health Science Center at San Antonio	homemade
H3K4me1	Abcam	ab8895
H3K4me3	Cell Signaling	#9727
H3K27Ac	Abcam	ab4729
H3K27me2	Cell Signaling	#9728
H3K27me3	Millipore Sigma	#07-449
Pol II	Santa Cruz	N-20
Pol II S5phosphorylation	Abcam	ab5131
rH2AX	millipore	clone JBW301
Normal Rabbit IgG	Cell Signaling	#2729
Donkey anti-Rabbit IgG (H+L) Highly Cross-Adsorbed Secondary Antibody, Alexa Fluor^™^ 546	ThermoFisher Scientific	A10040
Donkey anti-Mouse IgG (H+L) Highly Cross-Adsorbed Secondary Antibody, Alexa Fluor^™^ Plus 488	ThermoFisher Scientific	A32766
HRP-conjugated secondary antibody	Jackson ImmunoResearch laboratories	
Chemicals, peptides, and recombinant proteins		
pA/G-Tn5	This paper	N/A
Formaldehyde solution	Sigma-Aldrich	Cat#F8775
cOmplete EDTA-free Protease Inhibitor Cocktail	Sigma-Aldrich	Cat#11873580001
Proteinase K	Sigma-Aldrich	Cat#03115844001
RNase A	Thermo Fisher Scientific	Cat#12091021
TRIzol	Thermo Fisher Scientific	Cat#15596018
DMEM, high glucose, pyruvate	GIBCO	Cat# 11995065
Fetal bovine serum	VWR	Cat# 9706
Penicillin-Streptomycin	GIBCO	Cat# 15140122
Polybrene	Sigma-Aldrich	Cat# 107689-10G
Puromycin dihydrochloride	Thermo Fisher Scientific	Cat# A1113803
Q5 DNA Polymerase	New England Biolabs	Cat# M0491L
Dynabeads Protein G	Thermo Fisher Scientific	Cat# 10002D
LB Broth	Mp Biomedicals	Cat# 244610
L-Broth Agar Large Capsules	Mp Biomedicals	Cat# MP 113001236
2-Mercaptoethanol	Sigma Aldrich	Cat# M6250-10ML
VeriQuestTM Fast SYBRTM Green qPCR Master Mix (2×)	Thermo Fisher Scientific	Cat# C-756901ML
Polyjet *in vitro* DNA transfection reagent	Signagen	Cat# SL100688
EGS (ethylene glycol bis(succinimidyl succinate))	Thermo Fisher Scientific	Cat# 21565
MTT (3-(4,5-Dimethylthiazol-2-yl)-2,5-Diphenyltetrazolium Bromide)	Thermo Fisher Scientific	Cat# M6494
KOD Xtreme^™^ Hot Start DNA Polymerase	Sigma-Aldrich	Cat# 71975
Doxycycline hyclate	Millipore sigma	Cat# D9891
Critical commercial assays		
SuperScript^™^ III Reverse Transcriptase	Thermo Fisher Scientific	Cat# 18080093
Quick-RNA Miniprep	Zymo Research	Cat# R1054
ZymoPURE Plasmid Miniprep Kit	Zymo Research	Cat# D4211
QIAquick PCR Purification Kit	QIAGEN	Cat# 28104
QIAquick gel extraction kit	QIAGEN	Cat# 28704
TruSeq RNA Library Prep Kit v2	Illunina	Cat# RS-122-2001
Rapid DNA Lib Prep kit	Abclonal	Cat# RK20200
Mag-Bind^®^ TotalPure NGS beads	Omega	Cat# M1378-01
FITC Annexin V Apoptosis Detection Kit I	BD Pharmingen^™^	Cat# 556547
Qubit dsDNA HS Assay Kit	Thermo Fisher Scientific	Cat# Q32854
Deposited data		
PRO-seq	This paper	GSE264374
Cut&Tag	This paper	GSE264275
Experimental models: Cell lines		
GSC3028	Derived by Jeremy N. Rich laboratory	
GSC2907	Derived by Jeremy N. Rich laboratory	
GSC3565	Derived by Jeremy N. Rich laboratory	
GSC28	Derived by Jeremy N. Rich laboratory	
Mia PaCa-2	ATCC	CRL-1420
Experimental models: Organisms/strains		
Mouse:NSG mice (NOD.Cg-Prkdcscid Il2rgtm1Wjl/SzJ)	The Jackson Laboratory	JAX: 005557
Recombinant DNA		
pMD2.G	Addgene	RRID:Addgene_12259
psPAX2	Addgene	RRID:Addgene_12260
teton pLKO-shMed30	this paper	target: GGATCAATTACGAAATCTCAT
pTEPRT-MED30	this paper	N/A
pTEPRT-MED30-3XHA	this paper	N/A
pTEPRT-*c*-Myc-3XHA	this paper	N/A
shMED15_1	Sigma-Aldrich	TRCN0000018973
shMED15_2	Sigma-Aldrich	TRCN0000018969
shMED15_3	Sigma-Aldrich	TRCN0000275100
shMED15_4	Sigma-Aldrich	TRCN0000018970
shMED15_5	Sigma-Aldrich	TRCN0000275151
shMED30_1	Sigma-Aldrich	TRCN0000060725
shMED30_2	Sigma-Aldrich	TRCN0000060723
shMED30_3	Sigma-Aldrich	TRCN0000060724
shMED30_4	Sigma-Aldrich	TRCN0000060727
shMED30_5	Sigma-Aldrich	TRCN0000413310
shCtl	Sigma-Aldrich	catalog no. SCH0 02
Software and algorithms		
Trim Galore!	Galaxy Version 0.6.7+galaxy0	https://galaxy-main.usegalaxy.org/
Bowtie2	Galaxy Version 2.3.4.3+galaxy0	https://galaxy-main.usegalaxy.org/
bamCoverage	Galaxy Version 3.3.2.0.0	https://galaxy-main.usegalaxy.org/
MACS2 callpeak	Galaxy Version 2.1.1.20160309.6	https://galaxy-main.usegalaxy.org/
bedtools	Galaxy Version 2.30.0	https://galaxy-main.usegalaxy.org/
ChIPseeker	Galaxy Version 1.18.0+galaxy1	https://galaxy-main.usegalaxy.org/
HISAT2	Galaxy Version 2.2.1+galaxy0	https://galaxy-main.usegalaxy.org/
DESeq2	Galaxy Version 2.11.40.7+galaxy1	https://galaxy-main.usegalaxy.org/

## Data Availability

This paper does not report original code. High-throughput sequencing data have been deposited to GEO: GSE264374 and GSE264275. Any additional information required to reanalyze the data reported in this paper is available from the lead contact upon request.

## Highlights

- MED30 and MYC are frequently co-amplified genes across multiple cancer types
- MED30 redistributes MYC binding to its low-affinity regulatory regions
- MED30 overexpression is associated with *de novo* acquisition of H3K27ac-marked enhancers
- MED30-MYC cooperation drives transcriptional programs linked to tumor progression
