# Supplementary material for: Amplification of MED30 at chromosome 8q24 reprograms MYC binding to low-affinity oncogenic enhancers in cancer cells
Source: Cell Rep. Author manuscript; Available in PMC 2026 Jul 20. (PMC13384758; doi:10.1016/j.celrep.2026.117498)
Supplement: 1 [file NIHMS2190737-supplement-1.pdf]

Cell Reports, Volume *45*

Supplemental information

Amplification of MED30 at chromosome 8q24 reprograms MYC binding to low-affinity oncogenic enhancers in cancer cells

Chunyu Jin, Linjie Zhao, Wubin Ma, Guofeng Zhao, Yujia Liu, Hanwen Zhang, Shenghong Ma, Likun Yao, Yuan Liu, Qiulian Wu, Huairui Yuan, Kailin Yang, Wei Yuan, Kenneth Ohgi, Jeremy N. Rich, and Michael G. Rosenfeld

Supplemental Figure Legends

Figure S1. The genetic alterations of Mediator complex components in cancer patients.

(A) Examples of genetic alterations for all mediator components in cancer, samples with both mutation and CNV were searched (cBioPortal).
(B) Summary of major genetic alteration forms in cancer patients.

Figure S2. MED30- and MYC-regulated transcriptional program.

(A) PCA analysis of PRO-seq data: MYC overexpression (OE) vs Control, MED30 OE vs Control, MYC&MED30 double OE vs Control.
(B) Western blot validation of Tet-on MYC and/or MED30 overexpression Mia PaCa-2 cells.
(C) MYC mRNA expression level in RNA-seq in Dox-induced MED30 overexpression Mia PaCa-2 cells with or without 2 days of 0.5ug/ml Dox treatment (**FDR<0.01, n.s. FDR >0.05).
(D) Correlation of MED30 overexpress regulated genes PRO-seq vs. RNA-seq.

Figure S3. Genomic profiling of MED30 and other factors in control or MED30 overexpression condition.

(A) PCA plot of MED30 peak count density MED30 CUT&Tag with or without Dox-induced MED30 overexpression.
(B) Pie chart of MED30 peak distribution relative to gene.
(C) Transcription level change of gained MYC target genes (n=512, MYC bound enhancer less than 20kb of TSS) upon MED30 overexpression. A group of 500 random genes was used as control.
(D) Hallmark pathway enrichment of MED30 overexpression gained MYC target genes.
(E) Genome browser examples of MYC new generated bindings site (light blue highlighted) and nearby gene transcription in MED30 overexpression and control.
(F) Expression level of genes around new binding peak showed in (C), by RNA-seq (**p<0.01).
(G) H3K4me1 Hi-ChIP in human embryonic stem cell derived pancreatic hormone progenitor cells (H9/WA09) (ENCODE) visualization at HMGCL–FUCA and PURA–CYSTM1 gene loci.
(H) H3K4me1 ChIP-qPCR in indicated putative enhancer (enh) in Dox-induced Mia PaCa-2 cells Negative is a region that do not have H3K27ac peak.
(I) Western blot validation of Tet-on MED4 overexpression Mia PaCa-2 cells and qPCR test of the indicated MED30 target genes with or without Dox-induced MED4 overexpression.

Figure S4. MED30 is essential for the binding of other Mediator components on the genome.

(A) MED1 CUT&Tag tag density profile at the regions of MED30 gained or lost peaks.
(B) Genome browser snapshot of CUT&Tag results for MED30 and MED1 genome binding with or without MED30 overexpression in dox-induced MED30 overexpression Mia PaCa-2 cells.
(C) Genome browser snapshot of CUT&Tag results in the same loci of the upper panel for indicated factors in dox-induced shMED30 knockdown Mia PaCa-2 cells.

Figure S5. MED30 recruits other Mediator components and factors to the MED30 binding sites.

(A) Genome browser examples of indicated Mediator subunits and other factors on the sites of MED30 gained peaks in response to MED30 overexpression (CUT&Tag data of Tet-on MED30-3XHA Mia PaCa-2 cells with or without Dox)
(B) Genome browser tracks of histone marks with or without MED30 overexpression on random region, by CUT&Tag experiments in Tet-on MED30 overexpression Mia PaCa-2 cells.
(C) Tag density profile of indicated histone marks and transcription level (PRO-seq) on MED30 lost sites.
(D) Heatmap showing genome-wide H3K27ac mark peaks in control or MED30 overexpression condition.

Figure S6. Characterizing MED30 oncogenic properties in Mia PaCa-2 cells.

(A) Immunofluorescence (IF) images of rH2AX in doxycycline-induced shMED30 knockdown Mia PaCa-2 cells, and the percentage of rH2AX positive cells in random field of these two conditions. **$p<0.01$, two-tailed Student's t-test. Scale bar indicates 25um.

(B) Cell apoptosis detection in doxycycline-induced shMED30 knockdown Mia PaCa-2 cells with or without 0.5ug/ml dox for 3 days.

(C) qPCR validation of knockdown efficacy for shMED30 and shMED15 shRNAs used in Figure 6.

Figure S7. MED30 informs brain tumor grade and survival of cancer patients.

(A) Heatmaps of TCGA glioma RNA-seq samples (*n* = 308), displaying mRNA expression of Mediator component genes in addition to clinical and genetic variant information for each sample. NA, not applicable (http://gliovis.bioinfo.cnio.es/).

(B) Pearson correlation analysis of MED30 and MYC gene expression level in brain tumors CGGA dataset, separated by histology.

(C) Sample clustering for RNA-seq with indicated shRNA treatment in GSC3565 (by Deseq2).

(D) Volcano plot for differential expressed genes in RNA-seq of two MED30 shRNAs knockdown in GSC3565 cells (colored dots indicate genes of $|log2FC|>1$ and $FDR<0.05$).

(E) Pathway analysis of MED30-regulated geneset in GSC3565, using online "Enrichr" website.

(F) MED30 or MYC gene expression in human tissues reported by GTEx Analysis Release V8 (dbGaP Accession phs000424.v8.p2). Expression values are showed by TPM (transcripts per million) scaled by log Log10. Box plots are shown as median and 25th and 75th percentiles; points are displayed s outliers if they are above or below 1.5 times the interquartile range.

Figure S1

A

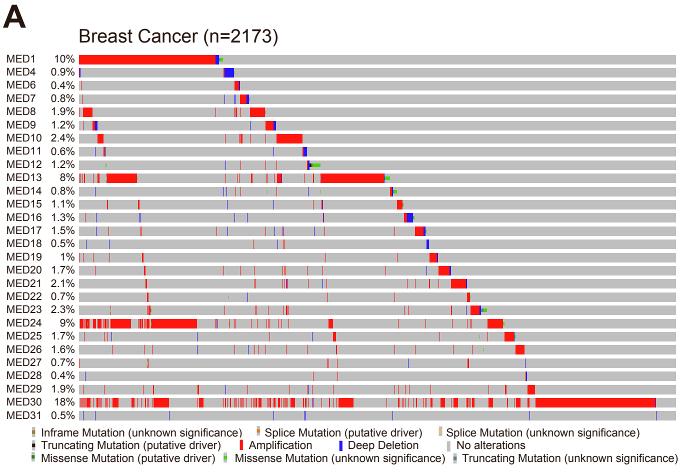

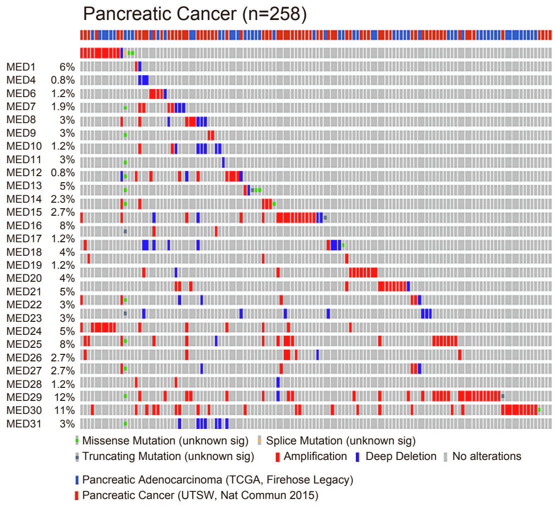

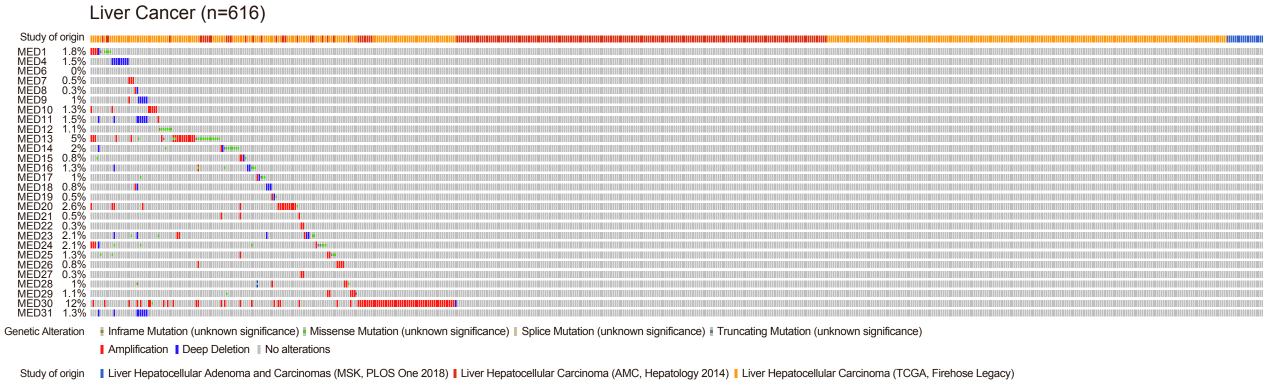

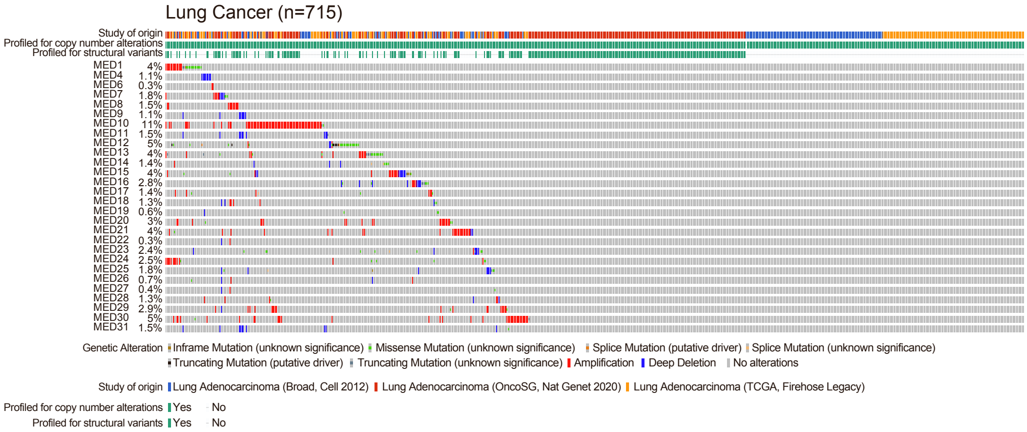

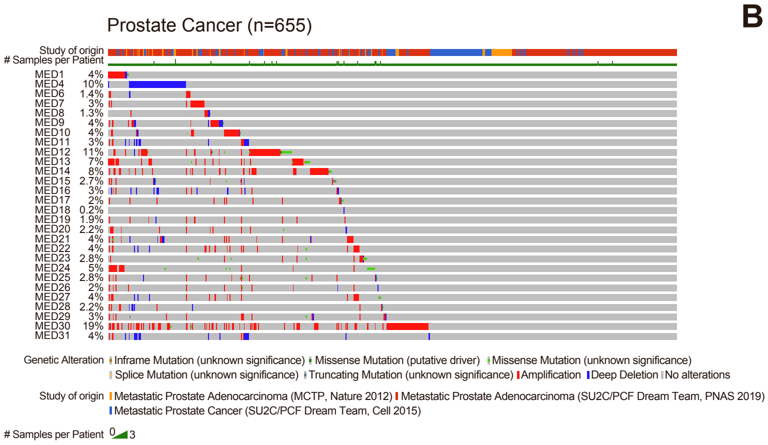

B

MED1	amp		
MED4		del	
MED6	less		
MED7	amp		
MED8	amp		
MED9	amp	del	
MED10	amp		
MED11		del	
MED12			mut
MED13	amp		
MED14	amp	del	mut
MED15	amp		
MED16	amp	del	
MED17	amp		
MED18	amp	del	
MED19	less		
MED20	amp		
MED21	amp		
MED22	amp		
MED23	amp		
MED24	amp		
MED25	amp		
MED26	amp		
MED27	amp		
MED28	amp		
MED29	amp		
MED30	amp		
MED31		del	

Figure S2

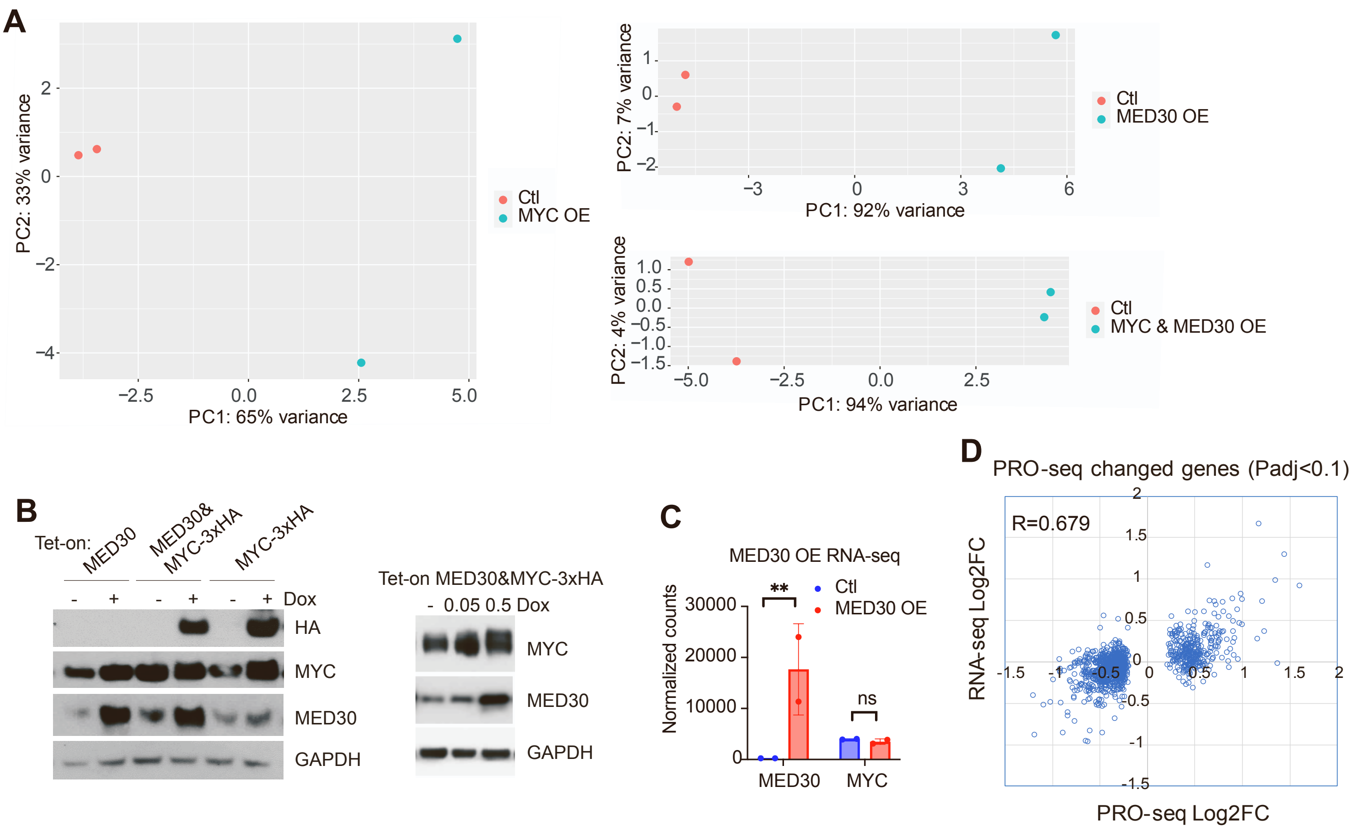

A
PC2: 33% variance
PC1: 65% variance
Ctl
MYC OE
PC2: 7% variance
PC1: 92% variance
Ctl
MED30 OE
PC2: 4% variance
PC1: 94% variance
Ctl
MYC & MED30 OE
B
Tet-on:
MED30
MED30&
MYC-3xHA
MYC-3xHA
Dox
HA
MYC
MED30
GAPDH
Tet-on MED30&MYC-3xHA
- 0.05 0.5 Dox
MYC
MED30
GAPDH
C
MED30 OE RNA-seq
Normalized counts
Ctl
MED30 OE
**
ns
MED30
MYC
D
PRO-seq changed genes (Padj<0.1)
R=0.679
RNA-seq Log2FC
PRO-seq Log2FC

Figure S3

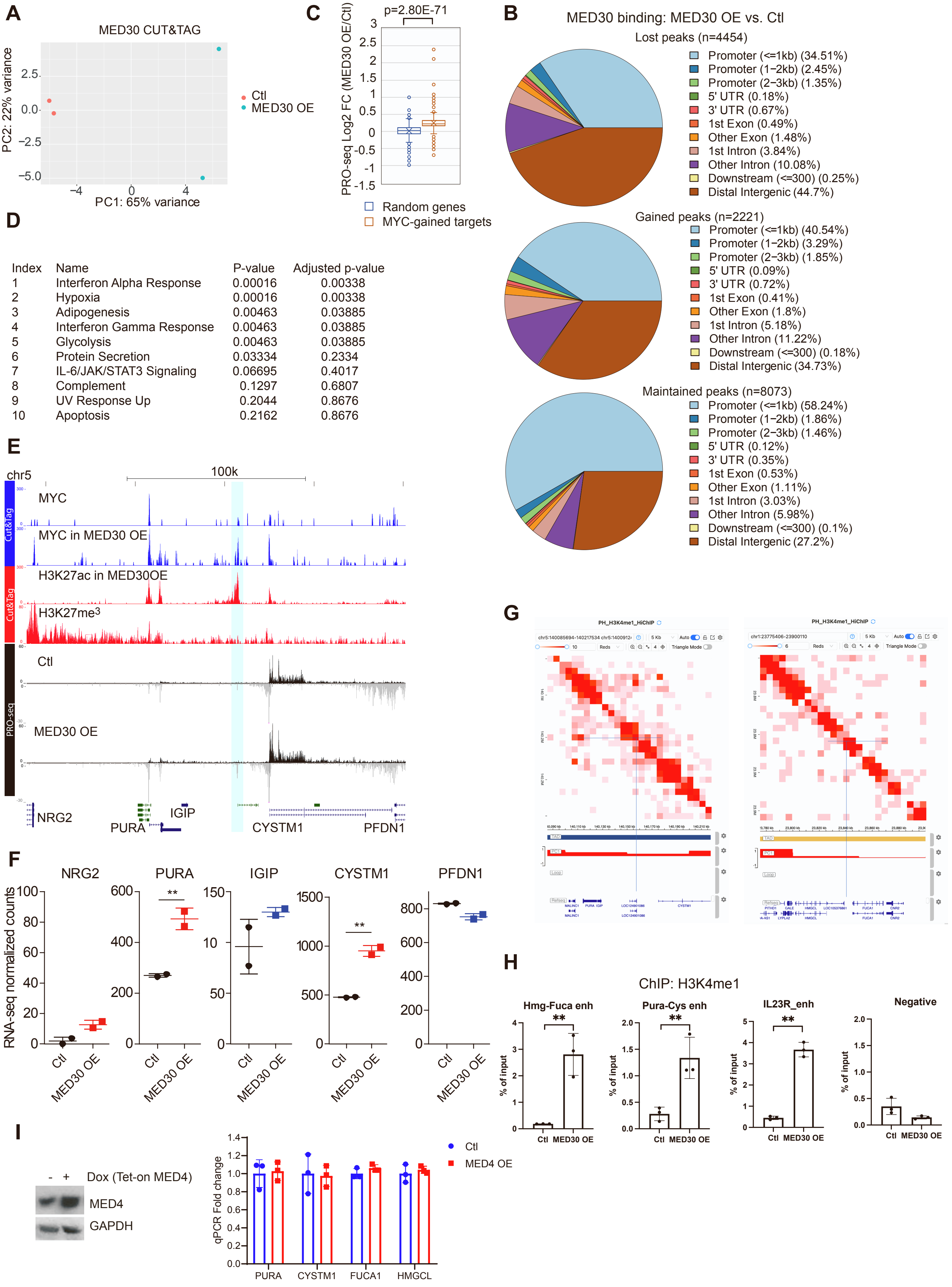
A
MED30 CUT&TAG
PC2: 22% variance
PC1: 65% variance
Ctl
MED30 OE
C
p=2.80E-71
PRO-seq Log2 FC (MED30 OE/Ctl)
Random genes
MYC-gained targets
B
MED30 binding: MED30 OE vs. Ctl
Lost peaks (n=4454)
Promoter (<=1kb) (34.51%)
Promoter (1−2kb) (2.45%)
Promoter (2−3kb) (1.35%)
5' UTR (0.18%)
3' UTR (0.67%)
1st Exon (0.49%)
Other Exon (1.48%)
1st Intron (3.84%)
Other Intron (10.08%)
Downstream (<=300) (0.25%)
Distal Intergenic (44.7%)
Gained peaks (n=2221)
Promoter (<=1kb) (40.54%)
Promoter (1−2kb) (3.29%)
Promoter (2−3kb) (1.85%)
5' UTR (0.09%)
3' UTR (0.72%)
1st Exon (0.41%)
Other Exon (1.8%)
1st Intron (5.18%)
Other Intron (11.22%)
Downstream (<=300) (0.18%)
Distal Intergenic (34.73%)
Maintained peaks (n=8073)
Promoter (<=1kb) (58.24%)
Promoter (1−2kb) (1.86%)
Promoter (2−3kb) (1.46%)
5' UTR (0.12%)
3' UTR (0.35%)
1st Exon (0.53%)
Other Exon (1.11%)
1st Intron (3.03%)
Other Intron (5.98%)
Downstream (<=300) (0.1%)
Distal Intergenic (27.2%)
D
Index Name P-value Adjusted p-value
1 Interferon Alpha Response 0.00016 0.00338
2 Hypoxia 0.00016 0.00338
3 Adipogenesis 0.00463 0.03885
4 Interferon Gamma Response 0.00463 0.03885
5 Glycolysis 0.00463 0.03885
6 Protein Secretion 0.03334 0.2334
7 IL-6/JAK/STAT3 Signaling 0.06695 0.4017
8 Complement 0.1297 0.6807
9 UV Response Up 0.2044 0.8676
10 Apoptosis 0.2162 0.8676
E
chr5
100k
MYC
MYC in MED30 OE
H3K27ac in MED30OE
H3K27me3
Ctl
MED30 OE
Cut&Tag
PRO-seq
NRG2
PURA
IGIP
CYSTM1
PFDN1
F
NRG2
PURA
IGIP
CYSTM1
PFDN1
RNA-seq normalized counts
Ctl
MED30 OE
G
PH_H3K4me1_HiChIP
H
ChIP: H3K4me1
Hmg-Fuca enh
Pura-Cys enh
IL23R_enh
Negative
% of input
Ctl
MED30 OE
I
Dox (Tet-on MED4)
MED4
GAPDH
qPCR Fold change
PURA
CYSTM1
FUCA1
HMGCL
Ctl
MED4 OE

Figure S4

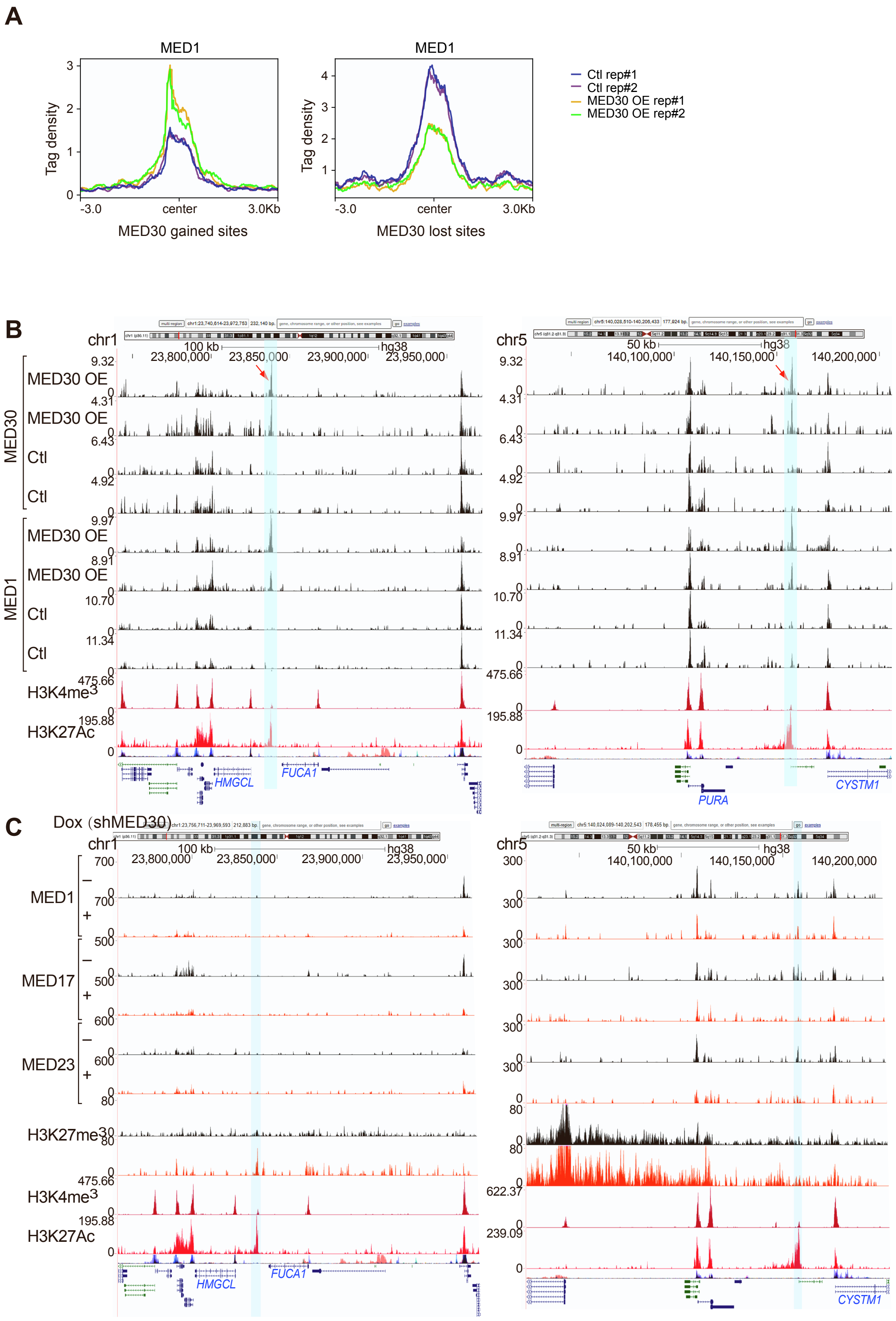
A
MED1
MED30 gained sites
MED30 lost sites
Tag density
-3.0
center
3.0Kb
Ctl rep#1
Ctl rep#2
MED30 OE rep#1
MED30 OE rep#2
B
chr1
chr5
MED30
MED1
MED30 OE
Ctl
H3K4me3
H3K27Ac
HMGCL
FUCA1
PURA
CYSTM1
C
Dox (shMED30)
MED1
MED17
MED23
H3K27me3
23,800,000
23,850,000
23,900,000
23,950,000
140,100,000
140,150,000
140,200,000
100 kb
50 kb
hg38

Figure S5

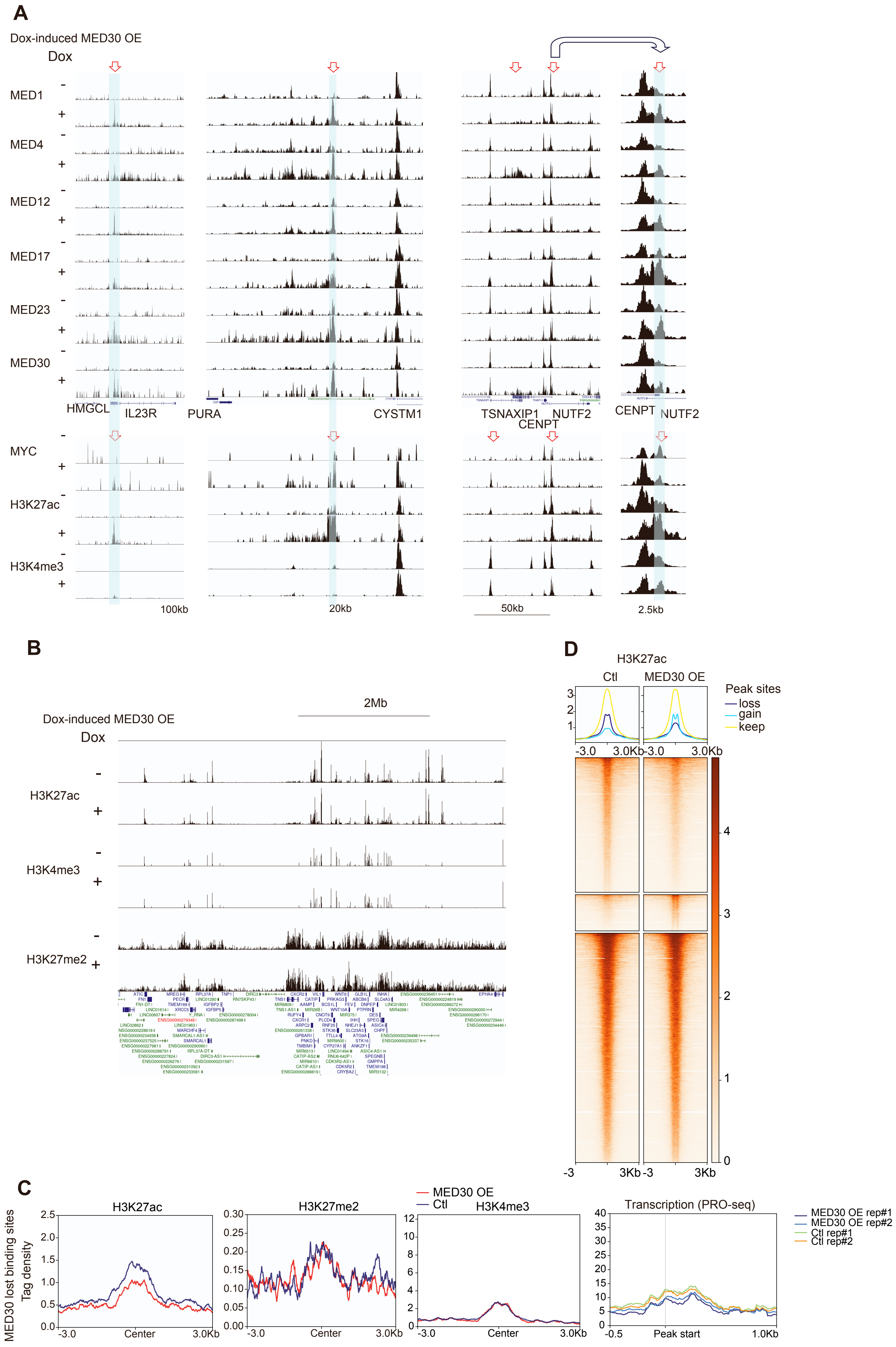

A
Dox-induced MED30 OE
Dox
MED1
MED4
MED12
MED17
MED23
MED30
HMGCL
IL23R
PURA
CYSTM1
TSNAXIP1
CENPT
NUTF2
CENPT
NUTF2
MYC
H3K27ac
H3K4me3
100kb
20kb
50kb
2.5kb
B
2Mb
Dox-induced MED30 OE
Dox
H3K27ac
H3K4me3
H3K27me2
D
H3K27ac
Ctl
MED30 OE
Peak sites
loss
gain
keep
-3.0
3.0Kb
-3
3Kb
C
MED30 lost binding sites
Tag density
H3K27ac
H3K27me2
MED30 OE
Ctl
H3K4me3
Transcription (PRO-seq)
MED30 OE rep#1
MED30 OE rep#2
Ctl rep#1
Ctl rep#2
-3.0
Center
3.0Kb
-0.5
Peak start
1.0Kb

Figure S6

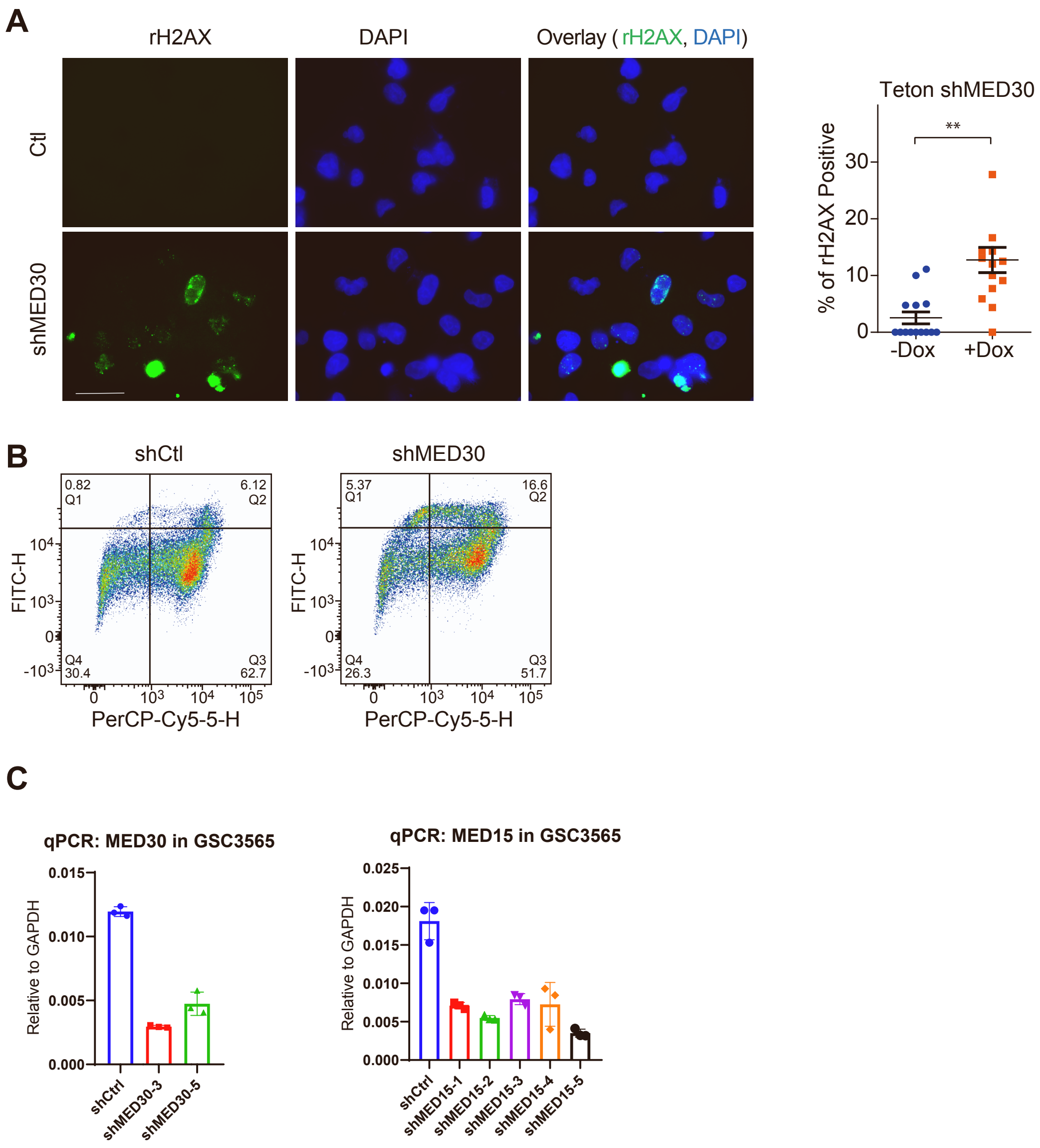
A
rH2AX
DAPI
Overlay (rH2AX, DAPI)
Ctl
shMED30
Teton shMED30
% of rH2AX Positive
**
-Dox
+Dox
B
shCtl
shMED30
0.82 Q1
6.12 Q2
Q4 30.4
Q3 62.7
5.37 Q1
16.6 Q2
Q4 26.3
Q3 51.7
FITC-H
PerCP-Cy5-5-H
C
qPCR: MED30 in GSC3565
qPCR: MED15 in GSC3565
Relative to GAPDH
shCtrl
shMED30-3
shMED30-5
shMED15-1
shMED15-2
shMED15-3
shMED15-4
shMED15-5

Figure S7

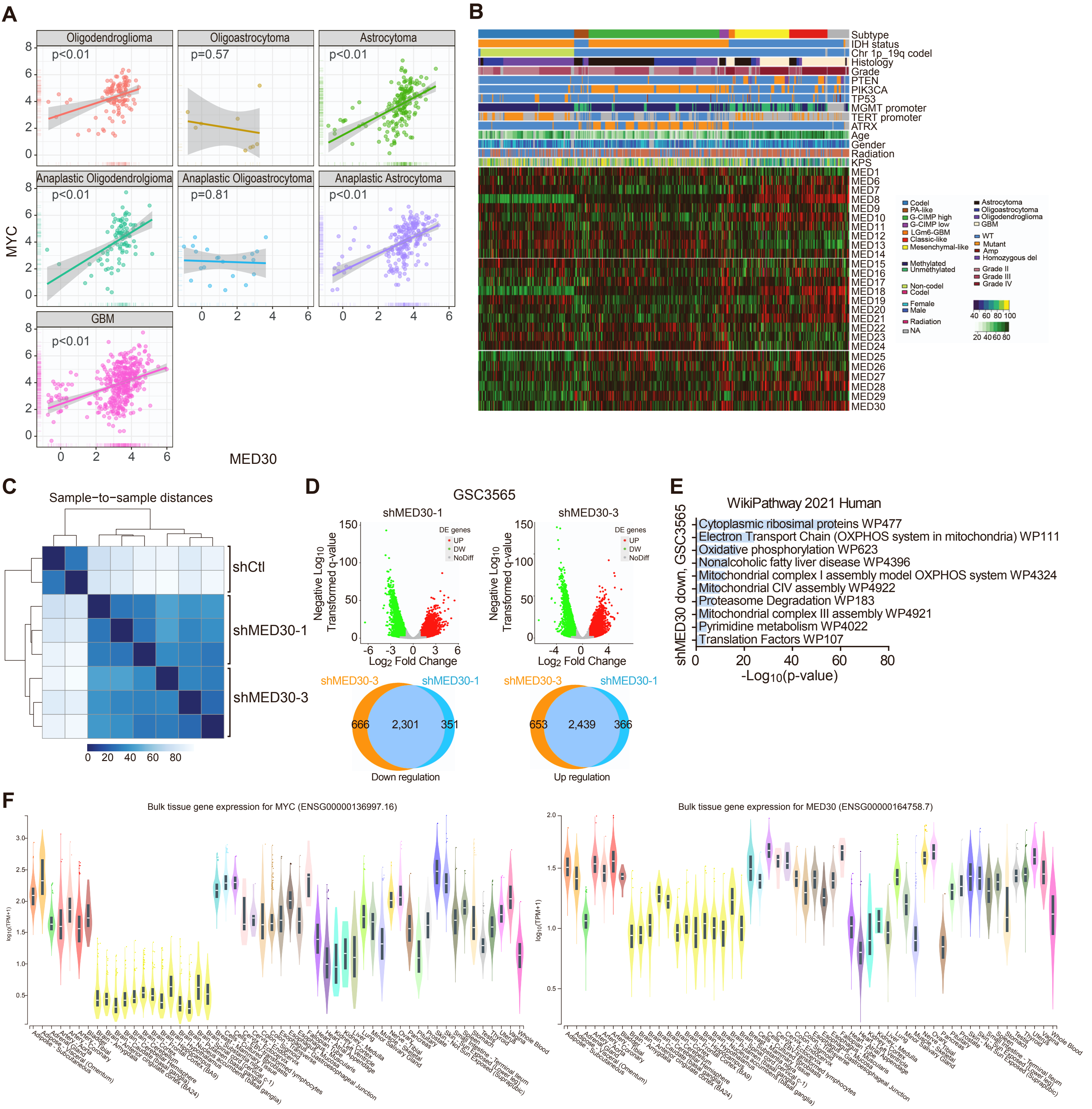
A
Oligodendroglioma
p<0.01
Oligoastrocytoma
p=0.57
Astrocytoma
p<0.01
Anaplastic Oligodendrolgioma
p<0.01
Anaplastic Oligoastrocytoma
p=0.81
Anaplastic Astrocytoma
p<0.01
GBM
p<0.01
MYC
MED30
B
Subtype
IDH status
Chr 1p_19q codel
Histology
Grade
PTEN
PIK3CA
TP53
MGMT promoter
TERT promoter
ATRX
Age
Gender
Radiation
KPS
MED1
MED6
MED7
MED8
MED9
MED10
MED11
MED12
MED13
MED14
MED15
MED16
MED17
MED18
MED19
MED20
MED21
MED22
MED23
MED24
MED25
MED26
MED27
MED28
MED29
MED30
Codel
PA-like
G-CIMP high
G-CIMP low
LGm6-GBM
Classic-like
Mesenchymal-like
Astrocytoma
Oligoastrocytoma
Oligodendroglioma
GBM
WT
Mutant
Amp
Homozygous del
Methylated
Unmethylated
Grade II
Grade III
Grade IV
Non-codel
Codel
Female
Male
Radiation
NA
C
Sample-to-sample distances
shCtl
shMED30-1
shMED30-3
D
GSC3565
shMED30-1
shMED30-3
DE genes
UP
DW
NoDiff
Negative Log10 Transformed q-value
Log2 Fold Change
shMED30-3
shMED30-1
666
2,301
351
Down regulation
653
2,439
366
Up regulation
E
WikiPathway 2021 Human
shMED30 down, GSC3565
Cytoplasmic ribosimal proteins WP477
Electron Transport Chain (OXPHOS system in mitochondria) WP111
Oxidative phosphorylation WP623
Nonalcoholic fatty liver disease WP4396
Mitochondrial complex I assembly model OXPHOS system WP4324
Mitochondrial CIV assembly WP4922
Proteasome Degradation WP183
Mitochondrial complex III assembly WP4921
Pyrimidine metabolism WP4022
Translation Factors WP107
-Log10(p-value)
F
Bulk tissue gene expression for MYC (ENSG00000136997.16)
Bulk tissue gene expression for MED30 (ENSG00000164758.7)
log10(TPM+1)
